# Rhamnose-containing glycans and lipids differentially contribute to the *Sporothrix*-host interaction

**DOI:** 10.3389/fmicb.2026.1901535

**Published:** 2026-07-20

**Authors:** Iván Martínez-Duncker, Joaquín O. Chávez-Santiago, Roberta Salinas-Marín, Manuela Gómez-Gaviria, Andréa Regina de Souza Baptista, Héctor M. Mora-Montes

**Affiliations:** 1 Laboratorio de Glicobiología Humana y Diagnóstico Molecular, Centro de Investigación en Dinámica Celular, Instituto de Investigación en Ciencias Básicas y Aplicadas, Universidad Autónoma del Estado de Morelos, Cuernavaca, Mexico; 2 División de Ciencias Naturales y Exactas, Departamento de Biología, Universidad de Guanajuato, Campus Guanajuato, Guanajuato, Mexico; 3 Center for Microorganisms' Investigation, Biomedical Institute, Fluminense Federal University, Campus Valonguinho, Rio de Janeiro, Brazil

**Keywords:** cell wall, glycolipid, phagocytosis, rhamnose, virulence

## Abstract

**Background:**

Rhamnose is a distinctive component of the cell wall of pathogenic *Sporothrix* species and plays important roles in host–pathogen interactions, including immune recognition, phagocytosis, and virulence. However, the mechanisms responsible for its incorporation into different surface glycoconjugates remain poorly understood. This study investigated the role of the putative rhamnosyltransferases *RHT1* and *RHT2* in *Sporothrix schenckii* and *Sporothrix brasiliensis*.

**Methods:**

*RHT1*- and *RHT2*-silenced mutants were generated in both species and analyzed for rhamnosyltransferase activity, cell wall composition, adhesion, biofilm formation, interactions with human immune cells, and virulence in the *Galleria mellonella* model. Cytokine production, TLR4-dependent recognition, and phagocytosis were evaluated to determine the contribution of distinct rhamnosylated glycoconjugates to host–pathogen interactions.

**Results:**

Silencing of either gene reduced total rhamnosyltransferase activity but produced distinct effects on cell wall glycoconjugates. *RHT2* silencing selectively decreased rhamnose incorporation into *N*-linked and *O*-linked glycans, whereas *RHT1* silencing primarily altered the carbohydrate composition of a surface glycolipid, indicating functional specialization. *RHT1*-silenced mutants displayed reduced adhesion to HeLa cells and impaired biofilm formation in both species. Immune interaction assays revealed species-specific effects: in *S. schenckii, RHT2* silencing had a stronger impact on cytokine production, TLR4-dependent recognition, and phagocytosis, whereas in *S. brasiliensis*, both *RHT1* and *RHT2* significantly contributed to these processes. Virulence assays showed that silencing either gene reduced virulence in *S. schenckii*, while virulence attenuation in *S. brasiliensis* was primarily associated with *RHT1* silencing.

**Conclusions:**

*RHT1* and *RHT2* perform specialized, non-redundant functions in the biosynthesis of rhamnosylated glycoconjugates in *Sporothrix*. The distribution of rhamnose between glycoproteins and glycolipids differentially influences immune recognition, host colonization, and virulence, revealing species-specific contributions to the pathogenicity of *S. schenckii* and *S. brasiliensis*.

## 1 Introduction

The genus *Sporothrix* groups fungal species with environmental, saprophytic, and entomopathogenic lifestyles (de Beer et al., 2016). Some of the species can grow and spread into mammalian tissues, causing sporotrichosis. *Sporothrix schenckii, Sporothrix brasiliensis, Sporothrix globosa*, and *Sporothrix luriei* belong to the pathogenic clade of *Sporothrix* and usually affect domestic species and human beings (de Beer et al., 2016; Dumont-Viollaz et al., 2025). Except for Antarctica, this infection has been found on all the continents, being considered a cosmopolitan disease (Gómez-Gaviria et al., 2025). However, the countries located in tropical areas usually report most of the sporotrichosis cases, and epidemic areas have been reported in Brazil, Peru, and Mexico (Chakrabarti et al., 2015). This paradigm is changing, as epidemic areas in China have been identified and outbreaks have been reported in South Africa (Govender et al., 2015; Cheng et al., 2024). Due to its identification more than a century ago, *S. schenckii* is the species most extensively studied so far (Lopes-Bezerra et al., 2018a,b), but *S. brasiliensis* is considered an emerging species of particular concern because of its high virulence and rapid spread in both humans and cats, causing outbreaks out of control in Brazil (Arrillaga-Moncrieff et al., 2009; Gómez-Gaviria et al., 2023a,b; Xavier et al., 2023).

Similar to other human fungal pathogens, except for *Cryptococcus* species, the *Sporothrix* cell wall is the outermost fungal structure that enters in contact with host components when invading tissues and organs (Gow, 2025). The cell wall composition, organization, and biogenesis are aspects of active research in fungal biology, because the knowledge generated in these areas is likely to contribute to the understanding of the host-fungus interaction and to finding new targets with diagnostic, therapeutic, and prophylactic potential (Díaz-Jiménez et al., 2012; Gow, 2025). The *S. schenckii* cell wall contains an inner layer composed of chitin and β-1,3-, β-1,4-, and β-1,6-glucans and a superficial layer of fibrillar glycoproteins with rhamnose-and mannose-containing glycoconjugates, which are known as peptidorhamnomannan (Lopes-Bezerra et al., 2018a,b). *S. brasiliensis* shows a similar cell wall structure and contains the same polysaccharides and glycoconjugates as *S. schenckii*, but with different proportions, containing more chitin and peptidorhamnomannan (Lopes-Bezerra et al., 2018a,b). Glycolipids are minor cell wall compounds in terms of abundance, but have relevant roles in cell signaling and interaction with the environment and the host (Beccaccioli et al., 2019). In *S. schenckii*, it was reported that cell wall glycolipids inhibit macrophage phagocytosis (Carlos et al., 2003); however, its diversity, abundance, and composition remain poorly studied. The monoclonal antibodies against the glycoinositol phosphorylceramide with the epitope Manpα1 → 3Manpα1 → 2IPC or glucosylceramide labeled the *S. schenckii* yeast-like cell wall, suggesting the presence of these molecules (Toledo et al., 2010). In addition, structural analyses identified glycoinositol phosphorylceramides substituted with mannose, mannobiose, mannotetraose, or mannosepentaose moieties in the *S. schenckii* cell wall (Penha et al., 2001).

Despite the fungal cell wall being rich in carbohydrates, rhamnose is an uncommon sugar found as part of the cell wall polysaccharides and glycoconjugates (Chávez-Santiago et al., 2025). It has currently been identified in the cell wall of *Sporothrix, Pseudallescheria boydii, Rhynchosporium secales, Cephalotheca purpurea, Cephalotheca reniformis, Penicillium chrysogenum, Ophiostoma ulmi, Magnaporthe grisea, Botryotinia fuckeliana, Botrytis cinerea*, and *Verticillium dahliae* (Figueiredo et al., 2010; Santhanam et al., 2017; Lopes-Bezerra et al., 2018a,b). However, bioinformatic analyses predict that this sugar is widely distributed in different fungal taxonomic groups, being elusive because of its low abundance in the cell wall (Chávez-Santiago et al., 2025). The role of rhamnose in the *S. schenckii*-host interaction has been studied in *RmlD*-silenced mutants, where the biosynthetic pathway of UDP-L-rhamnose synthesis was affected, and trace rhamnose levels were detected on the fungal cell wall (Tamez-Castrellón et al., 2021). These silenced mutants stimulated low TNFα and IL-6 levels in human peripheral blood mononuclear cells (PBMCs) and increased IL-1β and IL-10 production (Tamez-Castrellón et al., 2021). Moreover, the cells were more readily taken up by human monocyte-derived macrophages, suggesting a role in masking relevant cell surface components for the phagocytic process and showing virulence attenuation (Tamez-Castrellón et al., 2021). The use of these mutants helped to establish the recognition of cell wall rhamnose by the pattern recognition receptor TLR4 (Tamez-Castrellón et al., 2021). Regarding the biosynthetic machinery behind the synthesis of cell wall rhamnoglycoconjugates, two genes encoding rhamnosyltransferases, named *RHT1* and *RHT2*, were identified within the *S. schenckii* genome (Mora-Montes et al., 2022). Both are genes upregulated at 37 °C, and during interaction with the host (Mora-Montes et al., 2022). Currently, the information about cell wall rhamnose in *S. brasiliensis* is even more scarce, with reports only indicating its presence at the cell wall (Lopes-Bezerra et al., 2018a,b; Villalobos-Duno et al., 2021).

Here, to assess the contribution of *RHT1* and *RHT2* to the biology of *S. schenckii* and *S. brasiliensis*, fungal cells were transformed with a binary plasmid to silence either of these two genes, and the cell wall and host-fungus interaction were analyzed.

## 2 Materials and methods

### 2.1 Fungal strains and culture conditions

For *S. schenckii*, strain 1099-18 ATCC MYA was used as the parental strain for mutant generation, whilst for *S. brasiliensis*, the strain 5110 ATCC MYA 4823 was used. Both are referred to as the wild-type (WT) control strains, are from ATCC (https://www.atcc.org), and their genomes were already sequenced (Teixeira et al., 2014). Strains were transformed with one of the following constructs: pCambia-Nou, pCambia-Nou-*RHT1*, or pCambia-Nou-*RHT2*.

Fungal growth was performed in YPD plates [1% (w/v) yeast extract, 2 % (w/v) gelatin peptone, and 3 % (w/v) glucose, 2 %(v/v) agar], pH 4.5 at 28 °C, for 4 days. Conidia were harvested from agar surface and used for induction of yeast-like cells in YPD broth, pH 7.8, at 37 °C for 4 days, and orbital shaking at 120 rpm (Martínez-Álvarez et al., 2017). For selection and maintenance of mutant strains, the culture medium was supplemented with 25 μg mL^−1^ nourseothricin (GoldBio, St Louis, MO, USA).

### 2.2 Generation of binary plasmids and fungal transformation

For *RHT1* silencing, a 5′ end 397 bp fragment of the *S. schenckii RHT1* open reading frame (ORF; SPSK_05538 at https://www.ncbi.nlm.nih.gov/) was amplified with the primer pair5′ CTCGAGCGGTGTTTTGTGACTGTTGG3′ and5′ AAGCTTTGCCGAGTAAGTCTGGGTTC3′ (added recognition sites for XhoI and HindIII are underlined). This fragment is 96.5% identical to the same region in *S. brasiliensis RHT1* (SPBR_09002 at https://www.ncbi.nlm.nih.gov/). The amplicon was cloned into the pSilent-1 XhoI and HindIII sites (Nakayashiki et al., 2005), obtaining pSilent-1-*RHT1*-sense. The antisense region was generated by PCR with primers containing the same sequences that align within *RHT1*, but with adapter sequences for StuI and BglII. The amplicon was cloned into the StuI and BglII sites of pSilent-1-*RHT1*-sense, generating the pSilent-1-*RHT1*-sense-antisense. The same strategy was used to generate pSilent-1-*RHT2*-sense-antisense. In this case, a 5′ end 357 pb amplicon from *S. schenckii RHT2* (SPSK_01110) was synthesized by PCR with the primer pair 5′ CTCGAGGACGAACACATCCACGTCATCCA3′ and5′ AAGCTTCGACAGGCGCGAGATGAAAAAGAC3′ (added recognition sites for XhoI and HindIII are underlined). This fragment is 98.9% identical to the same region in *S. brasiliensis RHT2* (SPBR_07010 at https://www.ncbi.nlm.nih.gov/). From these constructs, the region spanning from the promoter (PtrpC) to terminator (TtrpC) was amplified with the primers5′ CTGCAGATGCCAGTTGTTGTTCCCAGTGATC3′ and3′ GAGCTCCCTCTAAACAAGTGTACCTGTGCATT5′ (added SacI and PstI sites are underlined) and cloned into the SacI and PstI sites of pCambia-Nou (Tamez-Castrellón et al., 2018), generating pCambia-Nou-*RHT1* and pCambia-Nou-*RHT2*. *Agrobacterium tumefaciens* AGL-1 was transformed with these binary plasmids and used to perform *A. tumefaciens*-mediated transformation as reported (Lozoya-Pérez et al., 2018). Transformant colonies were subjected to five monoconidial passages and two rounds of dimorphism to yeast-like cells to eliminate non-transformed nuclei (Lozoya-Pérez et al., 2018). As a control, the WT strains were also transformed with the empty pCambia-Nou vector. In all cases, PCR was used to confirm the presence of the binary plasmid within the fungal genome, using the primer pair5′ TAAGAGAGGTCCGCAAGTAGATT3′ and5′ TTAGGGGGGCAGGCAGGGCATGC3′ that amplifies a fragment of the gene that confers resistance to nourseothricin (Tamez-Castrellón et al., 2021).

### 2.3 Quantitative PCR assays

Quantitative PCR (qPCR) assays were used to analyze both gene expression and the number of binary plasmid insertional events within the fungal genome. For gene expression, the reactions were preceded by a retrotranscription step (RT-qPCR). Briefly, total RNA was extracted from yeast-like cells using TRI reagent (Sigma-Aldrich, St Louis, MO, USA), and oligo (dT)20-based cDNA synthesis and purification were performed as described (Trujillo-Esquivel et al., 2016). A Step One™ thermal cycler (Applied Biosystems, Waltham, MA, USA) was used for qPCR, using reactions containing 200 ng μL^−1^ cDNA, the same primer pair used for the generation of the binary plasmids (with no adaptor sequences added), and the SYBR Green PCR Master Mix kit (Applied Biosystems). All reactions produced a single amplicon, as judged by the analysis of melting curves. The amplification efficiency of primer pairs was 99.2% and 101.5% for primers amplifying part of the *RHT1* ORF from *S. schenckii* and *S. brasiliensis*, respectively, and 102.3% and 98.8% for the primer pair that amplifies a *RHT2* fragment from *S. schenckii* and *S. brasiliensis*, respectively. Relative expression levels were determined by calculating the 2^−Δ*Δct*^ (Livak and Schmittgen, 2001). Amplifications from the gene encoding the ribosomal protein L6 were used for data normalization (Trujillo-Esquivel et al., 2017). The primer pair used was 5′-ATTGCGACATCAGAGAAGG and 5′-TCGACCTTCTTGATGTTGG, and the amplification efficiency was 102.2% and 99.3% for *S. schenckii* and *S. brasiliensis*, respectively.

To calculate the number of binary plasmid insertional events, qPCR reactions with genomic DNA and the primer pair 5′-TAAGAGAGGTCCGCAAGTAGATT and 5′-TTAGGGGGGCAGGCAGGGCATGC were used. These primers amplify a fragment of the gene conferring resistance to nourseothricin (Tamez-Castrellón et al., 2018). The amplification efficiency of this primer pair was 98.6 %.

### 2.4 Enzyme activity assays

Yeast-like cells were grown for 4 days at 37 °C and 200 rpm, cells were harvested by centrifugation, and disrupted in an MSK cell homogenizer (Braun, Melsungen, Germany). Cells were broken with seven cycles of shaking for 1 min. Cell homogenates were centrifuged at 10,000 xg for 10 min at 4 °C, and the supernatant was saved and kept at −20 °C until use (Lopes-Bezerra et al., 2015). In some experiments, the homogenate was subjected to centrifugation at 100,000 × g for 1 h at 4 °C to separate the soluble and mixed membrane fractions. The rhamnosyltransferase activity was measured as reported (Yonekura-Sakakibara et al., 2007). Briefly, the reactions contained 400 ng α-1,6-mannobiose (Dextra Laboratories, Reading, UK), 500 μM UDP-L-rhamnose (Chemily Glycoscience; Peachtree Corners, GA), and 100 μg protein and were incubated for 1 h at 37 °C. Reactions were stopped by incubating at 100 °C for 10 min and were analyzed by High-Performance Anion-Exchange Chromatography with Pulsed Amperometric Detection (HPAEC-PAD) using a Dionex system (Thermo Fisher Scientific, Waltham, MA). The chromatographer was equipped with a CarboPac PA-100 column (4.6 × 250 mm), and carbohydrate separation was performed with a linear gradient of 10–100 mM sodium acetate in 100 mM NaOH using a flow rate of 0.8 mL min^−1^ for 30 min.

### 2.5 Cell wall analysis

Cell walls were obtained from yeast-like cells grown in YPD at pH 7.8 for 4 days at 37 °C and 200 rpm of reciprocal shaking (Martínez-Álvarez et al., 2017). Cells were harvested by centrifuging, washed three times with deionized water, and broken in a Braun homogenizer, as described above. The homogenates were centrifuged, the cell walls were pelleted, the supernatant was removed, and the walls were extensively incubated with NaCl, SDS, β-mercaptoethanol, and EDTA to remove contamination with intracellular proteins, as reported (Mora-Montes et al., 2007).

For sugar analysis, aliquots containing 5 mg of cell wall were hydrolyzed with 2 M trifluoroacetic acid at 100 °C overnight, followed by evaporation of the acid and resuspension in deionized water. Monosaccharides were separated and quantified by HPAEC-PAD, using a CarboPac PA-1 column (4.6 × 250 mm), using an isocratic gradient of 3.2 mM NaOH and a flow rate of 1.5 mL min^−1^ for 40 min (Plaine et al., 2008). For protein quantification, aliquots containing 10 mg cell wall were alkali-hydrolized in 1N NaOH, followed by neutralization with 1N HCl (Mora-Montes et al., 2007). The neutralized hydrolyzed was analyzed with the Pierce BCA protein assay (Thermo-Fisher Scientific).

Localization of the β-1,3-glucan and chitin within the cell wall was performed with the soluble dectin-1 and fluorescein-conjugated WGA, respectively, as reported (Martínez-Álvarez et al., 2017). For data normalization, cells were heat-inactivated at 60 °C for 2 h, and fluorescence signals from these cells were considered 100% of polysaccharide exposure at the cell wall surface (Martínez-Álvarez et al., 2017). Susceptibility to Congo Red, Calcofluor white, and SDS was performed as previously described (Lozoya-Pérez et al., 2019).

To analyze protein-linked oligosaccharides, 1 × 10^9^ cells mL^−1^ were subjected to deglycosylation as follows. To trim *N*-linked glycans, cells were suspended in 25 U of endoglycosidase H (New England Biolabs, Ipswich, MA, USA) and incubated for 18 h at 37 °C (Tamez-Castrellón et al., 2021). For *O*-linked glycan trimming, cells were suspended in 0.1 M NaOH and incubated for 24 h at 25 °C and 50 rpm (Tamez-Castrellón et al., 2021). In both treatments, cells were centrifuged, the supernatant was saved and used for glycan quantification by HPAEC-PAD, as described above.

For cell wall lipid extraction, 10 mg of dried cell wall was suspended in a solution of chloroform/methanol (2:1, v/v) and stirred for 2 h at 25 °C. Samples were centrifuged at 5,000 × g for 5 min at 4 °C; the pelleted material was saved and resuspended again in chloroform/methanol solution, and the extraction procedure was repeated once (Carlos et al., 2003). Then, the pelleted material was extracted twice with a solution of chloroform/methanol/water (10:10:1, v/v); the extracted material in each step was pooled and separated by partition in water and water-saturated-butanol (Trinel et al., 1999). The water phase was saved, lyophilized, and named as lipid extract. To remove the glycan moiety from the lipid extract, 10 mg of this was suspended in 1 N KOH and incubated for 90 min at 37 °C. Then, 1 N acetic acid was added to neutralize pH, the material was lyophilized, suspended in 0.1 M ammonium acetate, and separated in a Bio-Gel P2 (Bio-Rad, Hercules, CA, USA) as described (Trinel et al., 1999). Sugar elution was monitored with the modified phenol-sulfuric acid methodology, as reported (Yue et al., 2022). Standards for the mobility of a trisaccharide, disaccharide, and monosaccharide were maltotriose, maltose, and glucose, respectively (all from Sigma-Aldrich).

The cell wall lipid extract was subjected to enzymatic hydrolysis with the following enzymes: α-glucosidase from *Bacillus stearothermophilus* (Megazyme, Wicklow, Ireland), β-glucosidase from *Agrobacterium* sp. (Megazyme), β-N-acetylglucosaminidase from *Streptococcus pneumoniae* (Sigma-Aldrich), α-galactosidase from green coffee beans (Sigma-Aldrich), β-galactosidase from *Aspergillus niger* (Megazyme), α-L-rhamnosidase (Megazyme), β-mannosidase from *Helix pomatia* (Sigma-Aldrich), α-mannosidase from *Canavalia ensiformis* (Sigma-Aldrich), and α1,2-/α1,3-mannosidase (New England Biolabs). In all cases, the enzyme reactions were performed following the manufacturer's instructions. Alternatively, the cell wall lipid extract was subjected to acid hydrolysis with trifluoroacetic acid, as described above (Trinel et al., 1999). The released monosaccharides were analyzed by HPAEC-PAD as described for cell wall sugar analysis.

### 2.6 Adhesion to HeLa cells

The cell line HeLa (ATCC) was grown until reach a monolayer in Eagle's Minimum Essential Medium (EMEM, Sigma-Aldrich) at 37 °C and 5% CO_2_ (v/v). Cells were detached from the plastic surface and cell-cell junctions disrupted by incubating with 0.25% (w/v) trypsin (Sigma-Aldrich) and 0.53 mM EDTA (García-Carnero et al., 2021), washed twice with ice-cold PBS, concentration adjusted at 5 × 10^6^ cells mL^−1^, using a haemocytometer, and 1 × 10^6^ cells were seeded in Nunc MaxiSorp™ flat-bottom 96-well microplates (Sigma-Aldrich). These were incubated for 24 h at 37 °C and 5% (v/v) CO_2_, then 1 % (w/v) bovine serum albumin in PBS was added and further incubated for 2 h at 37 °C, and used in adhesion assays. Aliquots containing 5 × 10^6^ yeast-like cells in PBS were added, incubated for 1 h at 37 °C, washed with PBS-0.05% (v/v) Tween 20 (PBS-Tween), 100 μL of rabbit polyclonal anti-rHsp60 diluted at 1:3,000 was added, and incubated for 2 h at room temperature, washed with PBS-Tween, 100 μL of goat anti-rabbit IgG-peroxidase antibody diluted 1:5,000 (Sigma-Aldrich) was added and incubated for 2 h at room temperature. Wells were washed three times with PBS-Tween, and 0.1 mg mL^−1^ o-phenylenediamine dihydrochloride and 0.006 % (v/v) hydrogen peroxide were added, incubated at room temperature for 20 min, 50 μL 2 N sulfuric acid was added, and absorbance was read at 450 nm in a Varioskan LUX Multimode Microplate Reader (Thermo Fisher Scientific) (García-Carnero et al., 2021).

Similar experiments were performed with microplates coated with 1 μg of any of the following components of the extracellular matrix: human fibronectin, thrombospondin-1, type-I collagen, laminin, elastin, fibrinogen, or bovine type-II collagen (all from Sigma-Aldrich). The protocol was essentially as previously described (García-Carnero et al., 2021). Wells coated only with bovine serum albumin were included as controls.

### 2.7 Analysis of biofilm formation

Biofilm formation was assayed as described (Clavijo-Giraldo et al., 2023). Aliquots containing 1 × 10^6^ yeast-like cells were placed in flat-bottom Nunc polystyrene 96-microtiter plates (Thermo Fisher Scientific), incubated for 4 h at 37 °C, washed three times with PBS, 100 μL per well of RPMI-1640 medium supplemented with L-glutamine (Sigma-Aldrich) was added, and plates were incubated for 24 h at 37 °C. The wells were washed five times with PBS, 100 μL of neat methanol was added per well, plates were incubated for 15 min at room temperature, methanol was removed, 100 μL per well of 0.02% (w/v) crystal violet was added, incubated for 20 min at room temperature, and washed three times with deionized water. Finally, 150 μL per well of 33% (v/v) acetic acid was added, and the absorbance at 590 nm was read in a Varioskan LUX Multimode Microplate Reader (Thermo Fisher Scientific). Mock wells containing only RMPI-1640 medium were included as controls.

### 2.8 Ethical considerations

The use of insects and human samples was approved by the Institutional Research Ethics Committee of Universidad de Guanajuato (Ref. CEPIUG-P15-2023). Blood sample collection was conducted in accordance with the Declaration of Helsinki and performed only after adult and healthy volunteers agreed to participate and signed the corresponding informed consent. A total of eight human donors were enrolled in this study.

### 2.9 Isolation of human PBMCs and quantification of cytokine production

Human blood samples were obtained from venous puncture, using EDTA as an anticoagulant. PBMCs were enriched by differential centrifugation with Histopaque-1077 (Sigma-Aldrich), as reported (Endres et al., 1988). Cells were suspended in RPMI 1640 Dutch modification (added with 2 mM glutamine, 0.1 mM pyruvate, and 0.05 mg mL^−1^ gentamycin; all reagents from Sigma-Aldrich), cell concentration was adjusted to 5 × 10^6^ cells mL^−1^, aliquots of 100 μL per well were placed in round-bottom 96-well microplates, and 100 μL 1 x 10^6^ yeast-like cells per well were added. Plates were incubated for 24 h at 37 °C with 5 % (v/v) CO_2_, centrifuged for 10 min at 3,000 × g at 4 °C, and the supernatants were collected and used for cytokine measurement. In all plates, mock wells with either medium or only human PBMCs were included as controls. Cytokine levels measured from the control wells were subtracted from the values obtained from the cell-cell interactions. For IL-17 and IL-22 stimulation, yeast-like cells were inactivated by UV light, exposed to 4 doses of 100 mJ cm^2 − 1^ UV light in a UV-DNA crosslinker CL-3000 (Analytik Jena, Upland, CA, USA). The human PBMCs were supplemented with 10% (v/v) human pooled serum UV-killed cells added in the cell-cell proportion given above, and the plates were incubated for 7 days at 37 °C with 5% (v/v) CO_2_ (Tóth et al., 2013).

In some experiments, the human PBMCs were incubated, before the interaction with yeast-like cells, for 1 h at 37 °C and 5 % (v/v) CO_2_ with one of the following immune receptor antagonists: 200 μg mL^−1^ laminarin (Sigma-Aldrich), 10 μg mL^−1^ anti-mannose receptor (MR) (Thermo-Fisher Scientific), 10 μg mL^−1^ anti-TLR4 antibody (Santa Cruz Biotechnology, Dallas, TX, USA), 10 μg mL^−1^ anti-TLR2 antibody (Thermo-Fisher Scientific), and 10 μg mL^−1^ anti-CD11b antibody (CR3, Thermo-Fisher Scientific) (Martínez-Álvarez et al., 2017; Gómez-Gaviria et al., 2023a,b). As controls for the interactions in the presence of antibodies, PBMCs were preincubated with isotype-matched antibodies before interacting with fungal cells. The antibodies were 10 μg mL^−1^ IgG1 (Santa Cruz Biotechnology, control interaction with anti-TLR4 and anti-MR antibodies), 10 μg mL^−1^ of IgG2ak antibody (Thermo-Fisher Scientific, to control interactions with anti-TLR2 antibody), and 10 μg mL^−1^ of IgG2 antibody (R&D, Minneapolis, MN, USA, to control interactions with anti-CD11b antibody) (Martínez-Álvarez et al., 2017; Neves et al., 2021).

The IL-17, IL-22, and IL-1β were quantified by ELISA with a DuoSet ELISA Development kit from R&D Systems. IL-6, IL-10, and TNFα levels were measured with Standard ABTS ELISA Development kits (Preprotech) following the manufacturer's recommendations.

### 2.10 Analysis of phagocytosis by human monocyte-derived macrophages

Human PBMCs were isolated as described above and were incubated with recombinant human granulocyte-macrophage colony-stimulating factor (Sigma-Aldrich) for 6–7 days at 37 °C and 5% (v/v) CO_2_, as reported (Gómez-Gaviria et al., 2023a,b). Yeast-like cells were labeled with 1 mg mL^−1^ acridine orange (Sigma-Aldrich) for 30 min at room temperature. The unbound dye was removed by washing four times with PBS, and the cell concentration was adjusted to 3 × 10^7^ yeast cells mL^−1^ in PBS. Flat-bottom six-well microplates were used to perform the immune cell-fungal cell interaction, in 800 μL DMEM (Sigma-Aldrich), with a macrophage-yeast ratio of 1:6, and incubation at 37 °C for 2 h in a 5% CO2 (v/v) atmosphere (Hernández-Chávez et al., 2018). Plates were cooled at 4 °C for 5 min, cells detached with cold PBS and resuspended in 1.25 mg mL^−1^ trypan blue. The phagocytic process was analyzed in a FACSCanto II system (Becton Dickinson, Franklin Lakes, NJ, USA), collecting 50,000 events per sample and acquiring signals in the FL1 (green) and FL3 (red) channels, previously calibrated with human monocyte-derived macrophages without any labeling or interaction with yeast-like cells (Gómez-Gaviria et al., 2023a,b). Cells detected only in the red channel were categorized as being in the late stage of phagocytosis. In some experiments, the human monocyte-derived macrophages were preincubated with laminarin or blocking antibodies, essentially as described above.

### 2.11 Virulence assays in *Galleria mellonella*

Larvae were obtained from a local colony raised in the institutional insectary with a diet based on corn bran and honey, as reported (Clavijo-Giraldo et al., 2016). Criteria for larvae inclusion within the study were a 1.2–1.5 cm size, no body melanization, and active behavior. The insects were divided into groups containing 30 individuals, and this was the total number of hosts challenged with each fungal strain. Larvae were inoculated in the last left pro-leg with 1 x 10^5^ yeast-like cells and were under daily observation for 2 weeks. During this time, larvae were kept in Petri dishes at 37 °C, with chopped apple for hydration and daily removal of silk to avoid the entrance to the pupa stage. A group of 30 animals was inoculated only with PBS, and this was defined as the control group to assess the impact of both manipulation and the vehicle inoculated into the hemolymph. Larvae were considered dead when no movement was observed upon external stimuli and showed full body melanization. Upon recording the animal death or at the end of the observation period for those alive, hemolymph was collected, anticoagulated, and used to assess colony-forming units by serial dilutions in YPD plates, as reported. For analysis of hemolymph parameters, such as hemocyte levels, cytotoxicity, and phenol oxidase activity, groups of 30 larvae were inoculated with the same amount of yeast-like cells, incubated for 24 h at 37 °C and hemolymph was withdrawn and immediately used to quantify these parameters as described (García-Carnero et al., 2020; Lozoya-Pérez et al., 2020). For cytotoxicity, this was defined as the cell-free lactate dehydrogenase activity found in the hemolymph, and the 100 % activity was defined as the enzyme levels released from lysed hemocytes, as described (García-Carnero et al., 2020; Lozoya-Pérez et al., 2020).

### 2.12 Statistical analyses

The software GraphPad Prism 6 was used for statistical analysis of data. The Shapiro–Wilk test was used to determine whether the statistical tests to be used should be parametric or nonparametric. Data showing normality were analyzed with the *t*-test. Cytokine stimulation and phagocytosis were analyzed with the Mann–Whitney *U* test. Survival experiments are reported in Kaplan-Meier survival curves and were analyzed with the log-rank test. In all cases, statistical significance was set at *P* < 0.05.

## 3 Results

### 3.1 Generation of *Sporothrix schenckii* and *Sporothrix brasiliensis RHT1* and *RHT2* silenced mutants

*RHT1* and *RHT2* were identified as genes encoding rhamnosyltransferases in *S. schenckii*, with increased expression in yeast-like cells and interaction with the host (Mora-Montes et al., 2022). Even though no experimental characterization of these genes has thus far been performed in *S. brasiliensis*, it is predicted that this organism contains both genes, as the putative encoded proteins have 100% and 99% identity with *S. schenckii* Rht1 and Rht2 (Chávez-Santiago et al., 2025). Since gene silencing has proved to be a useful genetic tool to establish the relevance of genes and proteins within *S. schenckii* and *S. brasiliensis* biology (Padró-Villegas et al., 2025; Barros et al., 2026; Padró-Villegas et al., 2026), we generated the binary plasmids pCambia-Nou-*RHT1* and pCambia-Nou-*RHT2* and used them in *A. tumefaciens*-mediated transformation of both *S. schenckii* and *S. brasiliensis*. Using this strategy in three independent transformation rounds, we generated 578 and 458 mutant strains using pCambia-Nou-*RHT1* in *S. schenckii* and *S. brasiliensis*, respectively. For the case of transformation with pCambia-Nou-*RHT2*, 557 and 647 mutant strains were obtained in three transformation rounds, using *S. schenckii* and *S. brasiliensis*, respectively. Some of these mutants were randomly selected to carry out five monoconidial passages and two rounds of dimorphism to eliminate non-transformed nuclei (Lozoya-Pérez et al., 2018). Confirmation of the binary plasmid was performed by PCR (data not shown). Next, from these groups of mutants, the *RHT1* and *RHT2* expression was analyzed by RT-qPCR. Silencing levels ranged from 5% to 97% for both genes in the two fungal species. The insertional event of the binary plasmid is not site-directed, and consequently, ectopic integrations may lead to polar effects in the phenotype. Therefore, we randomly selected three strains with the maximum silencing levels for additional analyses. The consistency of phenotypes among the three strains would indicate that phenotypes are associated with gene silencing instead of the insertional site of the binary vector. The strains were named as HSS69, HSS70, and HSS71 for those where *RHT1* was silenced in *S. schenckii*; HSS72, HSS73, and HSS74 for *S. schenckii* mutants with *RHT2* silenced; HSB35, HSB36, and HSB37 for *S. brasiliensis* mutants with *RHT1* silenced, and HSB38, HSB39, and HSB40 for *S. brasiliensis* mutants with *RHT2* silenced (Table 1). As a control, the WT strains were transformed with the empty pCambia-Nou, and two mutant strains were randomly selected. The strains are HSS67 and HSS68 for *S. schenckii* and HSB28 and HSB29 for *S. brasiliensis* (Table 1). qPCR analysis, using a primer pair to amplify part of the gene conferring resistance to nourseothricin, was used to assess the number of insertional events of the binary plasmid within the fungal genome. All selected strains showed one copy of pCambia-Nou inserted within the genome (Table 1).

**Table 1:** Fungal strains used in this study.

Strain	Genotype	*RHT1* expression (%)^a^	*RHT2* expression (%)^a^	Binary vector copy number^b^
*Sporothrix schenckii*				
1099-18 ATCC MYA 4821	Wild-type (WT)	100.0 ± 0.0	100.0 ± 0.0	N.D.
HSS67	WT transformed with pCambia-Nou	98.7 ± 1.1	99.4 ± 0.2	1.1 ± 0.2
HSS68	WT transformed with pCambia-Nou	99.5 ± 1.2	99.8 ± 0.4	1.3 ± 0.3
HSS69	WT transformed with pCambia-Nou-*RHT1*	3.2 ± 0.6^*^	99.2 ± 0.8	0.8 ± 0.1
HSS70	WT transformed with pCambia-Nou-*RHT1*	2.8 ± 0.4^*^	98.2 ± 0.2	1.1 ± 0.4
HSS71	WT transformed with pCambia-Nou-*RHT1*	2.7 ± 0.2^*^	99.6 ± 0.6	1.2 ± 0.3
HSS72	WT transformed with pCambia-Nou-*RHT2*	98.4 ± 1.0	3.0 ± 0.5^*^	1.0 ± 0.2
HSS73	WT transformed with pCambia-Nou-*RHT2*	99.4 ± 1.1	2.5 ± 0.1^*^	0.8 ± 0.1
15.9-7.8,-1.4498.8ptHSS74	WT transformed with pCambia-Nou-*RHT2*	97.9 ± 2.4	2.8 ± 0.7^*^	0.9 ± 0.2
*Sporothrix brasiliensis*				
5110 ATCC MYA 4823	WT	100.0 ± 0.0	100.0 ± 0.0	N.D.
HSB28	WT transformed with pCambia-Nou	99.4 ± 0.2	99.0 ± 0.6	0.8 ± 0.1
HSB29	WT transformed with pCambia-Nou	98.9 ± 0.4	99.4 ± 0.7	1.3 ± 0.4
HSB35	WT transformed with pCambia-Nou-*RHT1*	2.7 ± 0.5^*^	99.5 ± 0.1	1.0 ± 0.2
HSB36	WT transformed with pCambia-Nou-*RHT1*	3.3 ± 0.7^*^	98.6 ± 0.3	1.1 ± 0.3
HSB37	WT transformed with pCambia-Nou-*RHT1*	2.9 ± 0.2^*^	99.5 ± 0.1	0.9 ± 0.2
HSB38	WT transformed with pCambia-Nou-*RHT2*	98.4 ± 0.5	3.4 ± 0.6^*^	1.3 ± 0.1
HSB39	WT transformed with pCambia-Nou-*RHT2*	99.7 ± 0.1	2.6 ± 0.5^*^	0.8 ± 0.3
HSB40	WT transformed with pCambia-Nou-*RHT2*	98.7 ± 0.4	2.8 ± 0.7^*^	1.1 ± 0.4

^a^Total RNA was extracted, cDNA was synthesized, and analyzed by RT-qPCR.

^b^Genomic DNA was isolated and analyzed by qPCR. In both essays, the gene encoding the ribosomal protein L6 was used for data normalization. Data are means ± SD of three independent experiments performed in duplicate. ^*^P < 0.05 when compared to WT, or control strains (HSS67 and HSS68 for *S. schenckii*; HSB28 and HSB29 for *S. brasiliensis*).

All the silenced strains showed cell and colony morphology similar to WT and control strains (data not shown). Yeast-like cells of silenced mutants showed a length and a width of 3.0 ± 0.3 μm and 0.5 ± 0.2 in average for both species, and these parameters were similar to those shown by the WT and control strains of both species (3.4 ± 0.2 μm and 0.7 ± 0.2 μm, for length and width, respectively). Regarding doubling times, the silenced mutant strains showed no significant changes when compared to WT or control strains from both species (3.2 ± 0.6 h for hyphae and 9.2 ± 0.6 h for yeast-like cells from silenced mutant strains on average vs. 2.9 ± 0.7 h for hyphae and 8.6 ± 0.8 h for yeast-like cells from WT and control strains from both species; *P* = 0.895). All the mutants generated in this study showed similar ability to undergo dimorphism as the WT and control strains (data not shown).

**Figure 1:**
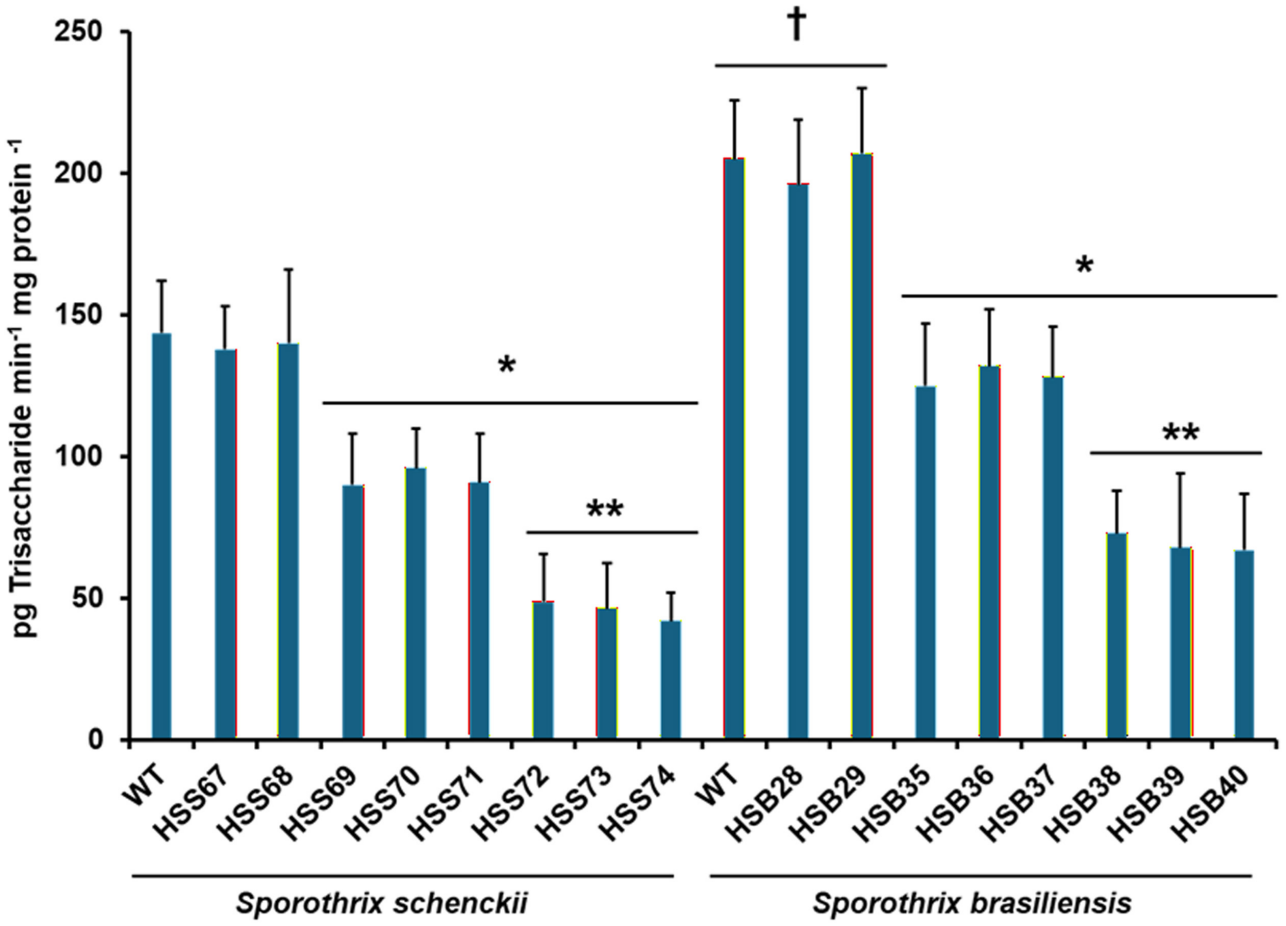
Rhamnosyltransferase activity in *Sporothrix schenckii* and *Sporothrix brasiliensis* homogenates. Yeast-like cells were mechanically disrupted and used to measure enzyme activity with α-1,6-mannobiose (acceptor) and UDP-L-rhamnose (donor). The synthesized trisaccharide was detected and quantified by high-performance anion-exchange chromatography with pulsed amperometric detection. Data are means ± SD of three biological replicates. Data normality was analyzed with Dunnett's test and significance with the T-test. *P < 0.05 when compared to WT or control strains (HSS67 and HSS68 for *S. schenckii* and HSB28 and HSB29 for *S. brasiliensis*). **P < 0.05 when compared to HSS69-HSS71 for *S. schenckii* or HSB35-HSB37 for *S. brasiliensis*. †*P* < 0.05 when compared to WT, HSS67 and HSS68 from *S. schenckii*. For *S. schenckii*, WT is strain 1099-18 ATCC MYA 4821. For *S. brasiliensis*, WT is strain 5110 ATCC MYA 4823.

Currently, it is unknown whether *RHT1* and *RHT2* belong to a gene family. This aspect is relevant because, in terms of functionality, gene family members usually have compensatory behaviors when one member is absent from the system (Peng, 2019; Flipphi et al., 2023). Thus, we measured the expression of the non-silenced gene in the mutants generated. The *RHT1*-silenced mutants from both species showed similar *RHT2* when compared to the WT and control strains from both species (Table 1). A similar result was observed in *RHT2*-silenced mutants from both species; this is *RHT1* expression closer to the levels shown by the WT and control strains (Table 1). We also measured the rhamnosyltransferase activity in all the strains under analysis. For both species, the *RHT1*-silenced strains showed a reduction in enzyme activity of about 35% when compared to WT and control strains, while *RHT2*-silenced mutants showed a reduction of about 70% in enzyme activity (Figure 1). It is noteworthy that no significant differences among the three strains showing silencing of one of the genes were observed (Figure 1). In addition, the WT and control strains showed higher enzyme activity in *S. brasiliensis* than in *S. schenckii*, and consequently, this observation was persistent in the silenced strains (Figure 1). When the soluble and the mixed membrane fraction were separated from the cell homogenate and used to assay enzyme activity, more than 97% of enzyme activity was associated with the mixed membrane fraction (data not shown).

### 3.2 Silencing of *Sporothrix schenckii* and *Sporothrix brasiliensis RHT1* or *RHT2* and changes in the cell wall

**Figure 2:**
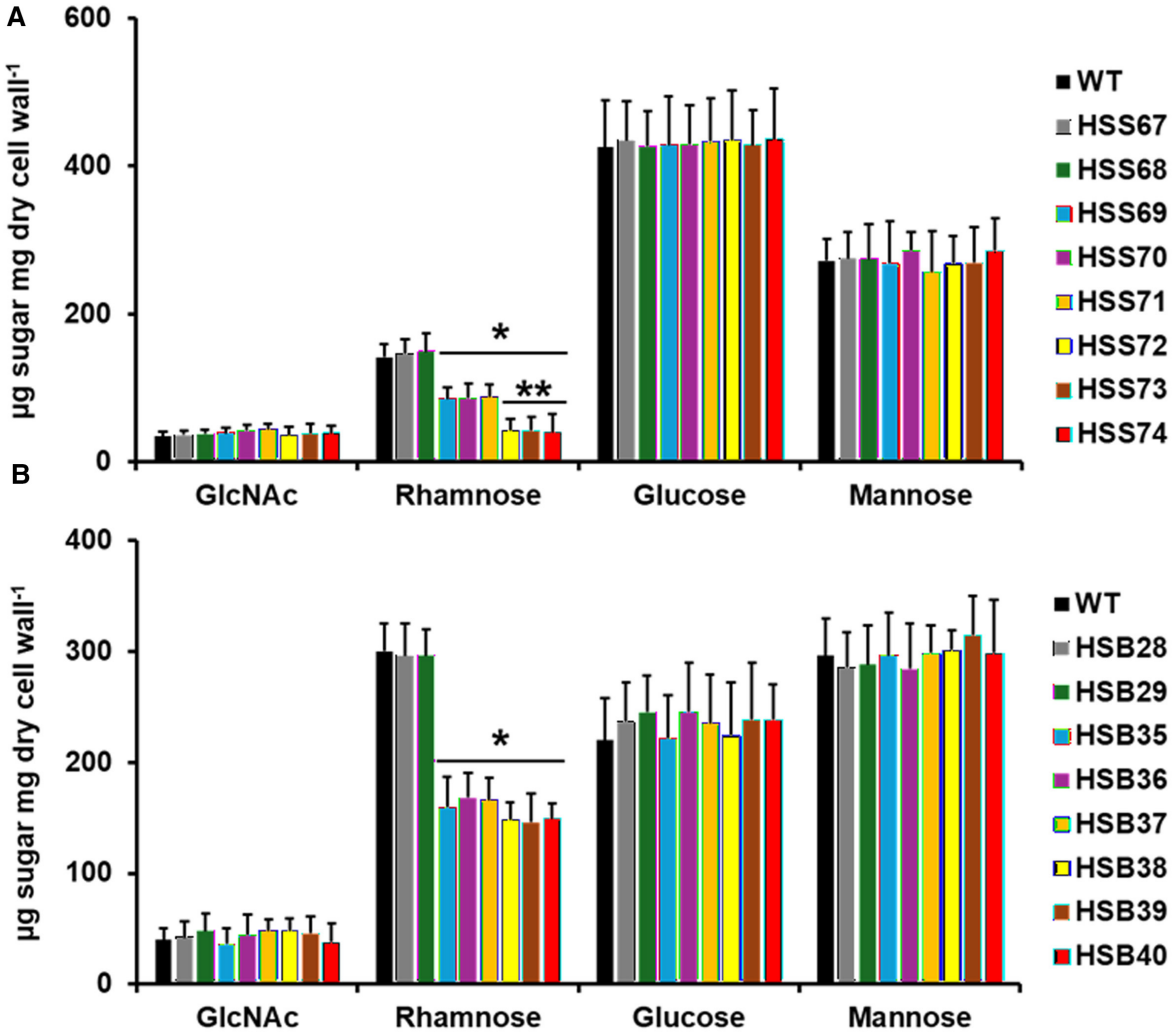
Analysis of cell wall monosaccharides of wild-type, control, and silenced mutant strains from *Sporothrix schenckii* and *Sporothrix brasiliensis*. Cell walls were isolated from yeast-like cells, acid-hydrolyzed to break glycosidic bonds, and the resulting sugars were separated and quantified by high-performance anion-exchange chromatography with pulsed amperometric detection. The results from *S. schenckii* strains are shown in **(A)**, whilst those from *S. brasiliensis* are in **(B)**. Results are shown as means ± SD of three biological replicates and were analyzed with Dunnett's test and then the *T*-test. *P < 0.05 when compared to WT or control strains (HSS67 and HSS68 for *S. schenckii*; HSB28 and HSB29 for *S. brasiliensis*). **P < 0.05 when compared to HSS69, HSS70 or HSS71. For *S. schenckii*, WT is strain 1099-18 ATCC MYA 4821. For *S. brasiliensis*, WT is strain 5110 ATCC MYA 4823. GlcNac, N-acetylglucosamine.

It was previously demonstrated that the *RmlD* silencing affected the cell wall rhamnose content in *S. schenckii* (Tamez-Castrellón et al., 2021). Therefore, it was likely that the *RHT1* or *RHT2* silencing affected cell wall rhamnose-containing glycoconjugates. The cell walls were acid hydrolyzed, and consequently, glycosidic bonds were disrupted in both polysaccharides and glycoconjugates, releasing N-acetylglucosamine (from chitin), glucose (from glucans), rhamnose, and mannose (from *O*-linked and *N*-linked glycans (Martínez-Álvarez et al., 2017). The *S. schenckii* WT and control strains (HSS67 and HSS68) showed a previously reported cell wall monosaccharide profile, which was glucose>mannose>rhamnose>N-acetylglucosamine (Lopes-Bezerra et al., 2018a,b; Figure 2). The *RHT1*- and *RHT2*-silenced mutants did not show changes in glucose, mannose, and N-acetylglucosamine when compared to the WT or control cells (Figure 2). However, all the silenced strains showed reduced rhamnose levels, which were significantly lower for the *RHT2*-silenced strains (HSS72-HSS74) when compared to the *RHT1*-silenced mutants (Figure 2). For *S. brasiliensis*, the WT and control strains (HSB28 and HSB29) showed monosaccharide profiles similar to those previously reported (Lopes-Bezerra et al., 2018a,b), with a profile of mannose> rhamnose> glucose> N-acetylglucosamine (Figure 2). Similar to *S. schenckii*, the *RHT1*- and *RHT2*-silenced mutants showed only a reduction in the rhamnose levels, which were similar for both sets of silenced mutants (HSB35-HSB37 and HSB38-HSB40, Figure 2).

**Figure 3:**
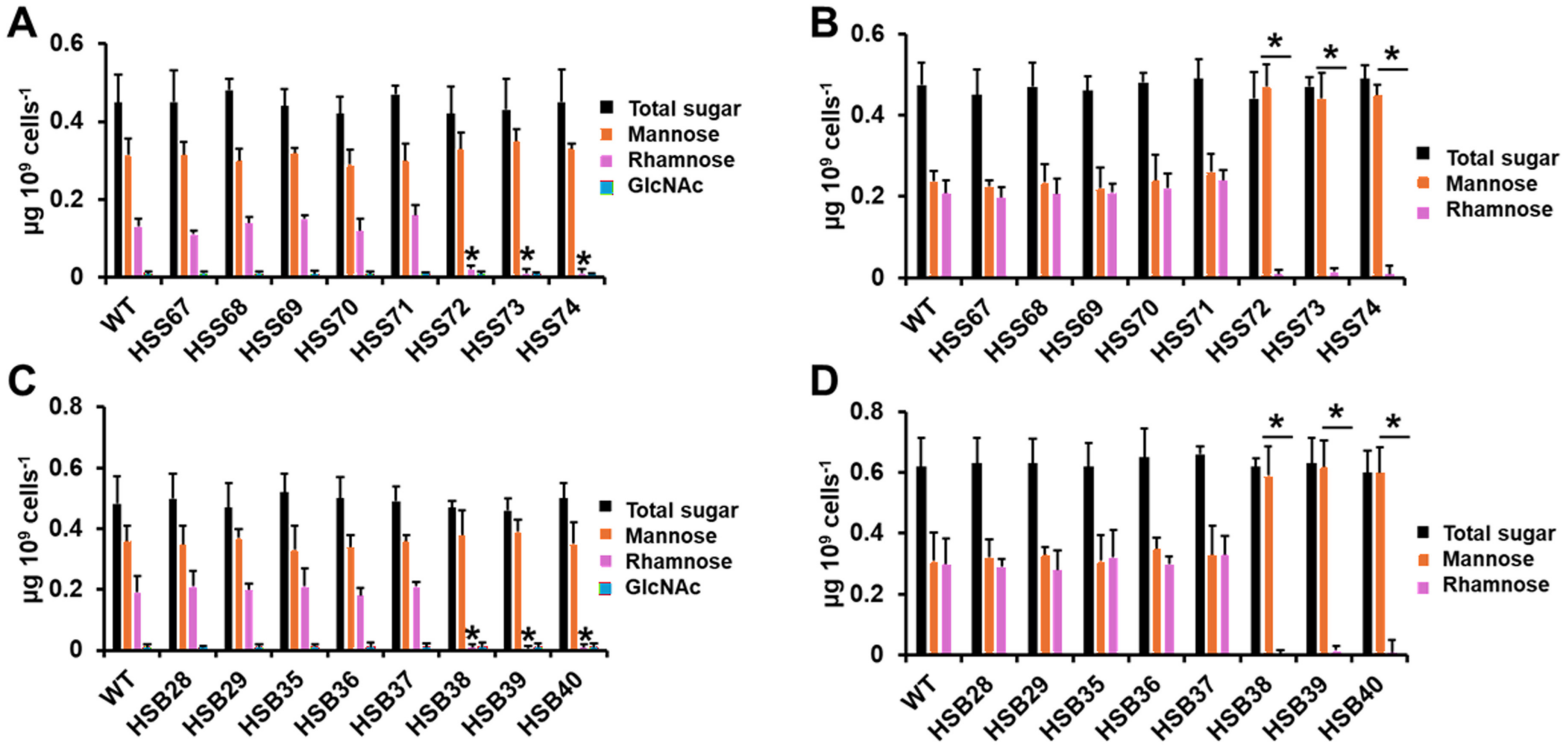
Analysis of *O*-linked and *N*-linked glycan from *Sporothrix schenckii* and *Sporothrix brasiliensis* cell walls. Aliquots containing 10^9^ yeast-like cells were incubated with either endoglycosidase H **(A, C)** or β-eliminated **(B, D)** to trim *N*-linked or *O*-linked glycans, respectively. The glycans were acid-hydrolyzed and analyzed by high-performance anion-exchange chromatography with pulsed amperometric detection. **(A, B)** are results with *S. schenckii* cells, whilst **(C, D)** show results generated with *S. brasiliensis* cells. Results are shown as means ± SD of three biological replicates and were analyzed with Dunnett's test and then the *T*-test. *P < 0.05 when compared to WT or control strains (HSS67 and HSS68 for *S. schenckii*; HSB28 and HSB29 for *S. brasiliensis*). For *S. schenckii*, WT is strain 1099-18 ATCC MYA 4821. For *S. brasiliensis*, WT is strain 5110 ATCC MYA 4823. GlcNac, N-acetylglucosamine.

The cell wall protein content did not show significant differences in all the analyzed strains (average in *S. schenckii* strains 182.6 ± 25.6 μg mg cell wall^−1^; average in the *S. brasiliensis* strains 228 ± 18.1 μg mg cell wall^−1^). None of the strains showed changes in the distribution of chitin and β-1,3-glucan whitin the cell wall, with similar exposure of both polysaccharides at the cell surface in WT, control and silenced mutants from both fungal species (average exposure of chitin and β-1,3-glucan in *S. schenckii*, 13.4 ± 6.6 % and 25.7 ± 10.4 %, respectively; average exposure of chitin and β-1,3-glucan in *S. brasiliensis*, 3.9 ± 2.7 % and 43.7 ± 14.5 %, respectively). All silenced strains showed susceptibility to Congo Red, Calcofluor White, and SDS similar to WT and control strains (data not shown). Next, we analyzed the *N*-linked glycan and *O*-linked glycan content at the cell wall of the *S. schenckii* and *S. brasiliensis* strains. Yeast-like cells were incubated with endoglycosidase H or β-eliminated to release *N*-linked or *O*-linked glycans, respectively, and sugars were quantified by HPAEC-PAD (Tamez-Castrellón et al., 2021). The WT, control, *RHT1*- and *RHT2*-silenced mutants from both fungal species showed similar *N*-linked and *O*-linked levels (Figure 3). For the case of *N*-linked glycans, both species showed mannose as the main component, followed by rhamnose and N-acetylglucosamine. The latter was found in trace amounts (Figure 3). In both *S. schenckii* and *S. brasiliensis, N*-linked glycans were rhamnose-depleted in the *RHT2*-silenced mutants, while the *RHT1*-silenced mutants showed rhamnose levels similar to those observed in the WT and control strains (Figure 3). For *O*-linked glycans, a similar trend was observed: no changes in rhamnose content in *RHT1*-silenced mutants and a significant reduction in the presence of this monosaccharide in the oligosaccharides from *RHT2*-silenced strains (Figure 3).

**Figure 4:**
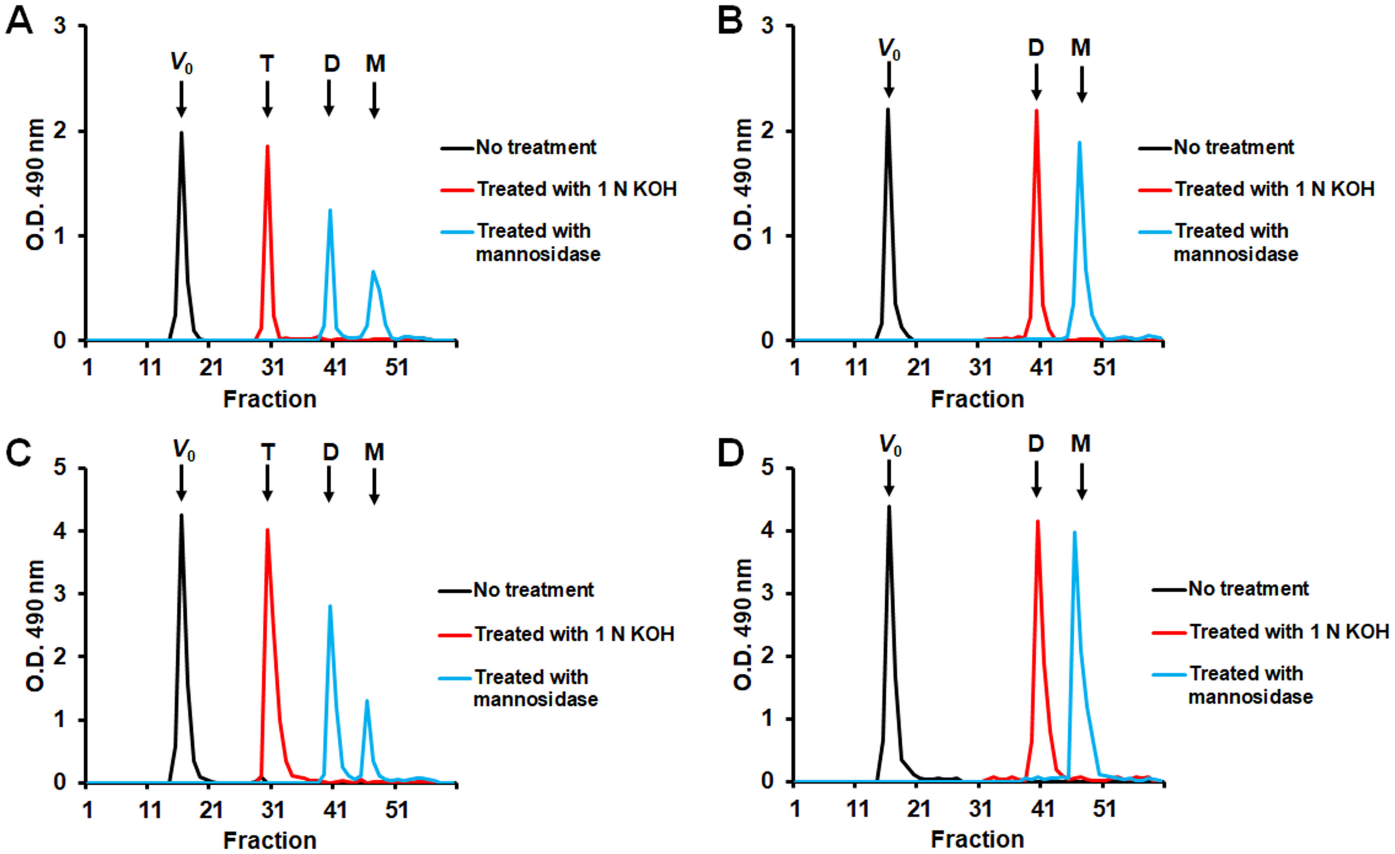
Analysis of cell wall glycolipids from *Sporothrix schenckii* and *Sporothrix brasiliensis* cell walls. Cell walls were isolated from yeast-like cells, and glycolipids were extracted with chloroform and methanol as described in the Materials and Methods. The lipid extract was either separated by size-exclusion chromatography in a Bio-Gel P2 column (No treatment) or alkali-hydrolyzed to release the glycoconjugate moiety (Treated with 1 N KOH). Alternatively, the alkali-released material was digested with *Canavalia ensiformis* α-mannosidase (Treated with mannosidase). Representative chromatograms of samples from *S. schenckii* and *S. brasiliensis* WT strains are shown in **(A, C)**, respectively. **(B, D)** show representative chromatograms generated with samples from *S. schenckii* and *S. brasiliensis RHT1*-silenced strains, respectively. *V*_0_, void volume; T, D, and M indicate the elution of molecular markers for a trisaccharide, disaccharide, and monosaccharide, respectively. For *S. schenckii*, WT is strain 1099-18 ATCC MYA 4821, and the *RHT1*-silenced strain is HSS70. For *S. brasiliensis*, WT is strain 5110 ATCC MYA 4823, and the *RHT1*-silenced strain is HSB36.

As mentioned, the *S. schenckii* cell wall contains glycolipids (Carlos et al., 2003). So, we hypothesize whether these components may also contain rhamnose, similar to cell wall glycoproteins. Cell wall lipids were extracted from walls with chloroform and methanol, following a previously reported protocol (Carlos et al., 2003), and the glycan moiety was removed by hydrolysis with 1 N KOH (Trinel et al., 1999). The cell wall lipid extract from *S. schenckii* and *S. brasiliensis* WT, control, and silenced strains was separated by size-exclusion chromatography in a Bio-gel P2 column and eluted with the void volume (Figure 4). For the case of WT and control strains from broth species, the hydrolysis with alkali released a trisaccharide (Figure 4 and data not shown). This trisaccharide was also obtained from the glycolipid extracted from the *RHT2*-silenced strains from both species (data not shown). For both *S. schenckii* and *S. brasiliensis RHT1*-silenced mutants, a disaccharide was trimmed from the glycolipid extract, instead of the trisaccharide observed in preparation from the WT cell wall (Figure 4). The hydrolyzed glyoconjugate moiety of this glycolipid was treated with different glycosidases and showed resistance to hydrolysis with α-glucosidase, β-glucosidase, β-N-acetylglucosaminidase, α-galactosidase, and β-galactosidase, suggesting that it did not contain glucose, galactose, or N-acetylglucosamine (data not shown). The hydrolysis of the trisaccharide from WT, control, and *RHT2*-silenced mutants from both species was digested by α-mannosidase from *Canavalia ensiformis*, generating a disaccharide and a monosaccharide (Figure 4 and data not shown). However, the trisaccharide was not digested by an α1,2-/α1,3-mannosidase (data not shown). Since the enzyme from *Canavalia ensiformis* has activity against α1,2-, α1,3-, and α1,6- linkages (Gnanesh Kumar et al., 2014), the results suggested the presence of an α1,6-mannobiose group within the trisaccharide structure. Furthermore, the trisaccharide digested with α-L-rhamnosidase generated a disaccharide and a monosaccharide, suggesting the presence of rhamnose within the structure of this glycoconjugate (data not shown). For the case of the disaccharide from *RHT1*-silenced strains from both species, these were not digested with any of the tested glycosylhydrolases, except for the α-mannosidase from *Canavalia ensiformis* (Figure 4).

To confirm these results, the glycan moiety of the cell wall glycolipid was acid-hydrolyzed, and HPAEC-PAD was used to analyze the monosaccharide content. The WT, control, and *RHT2*-silenced mutants from both *S. schenckii* and *S. brasiliensis* showed a similar monosaccharide composition, which contained mannose and rhamnose in a proportion of 2:1, respectively (Table 2). The samples from the *RHT1*-silenced mutants (HSS70-HSS72, and HSB35-HSB37) showed similar mannose content as the WT and control strain but trace rhamnose levels (Table 2). In all cases, the sugar content was not significantly modified when quantified from the whole glycolipid (Table 2). It is noteworthy to mention that the monosaccharide content in the *S. brasiliensis* samples was significantly higher than the levels observed in the *S. schenckii* strains (*p* < 0.05 in all cases; Table 2). Collectively, these data suggest that *RHT1*-silencing selectively affected the glycolipid synthesis, whose glycan moiety is Man-α-1,6-Man-α-Rhamnose, while silencing of *RHT2* only affected protein glycosylation in both *S. schenckii* and *S. brasiliensis*.

**Table 2:** Monosaccharide content of cell wall glycolipid from *Sporothrix schenckii* and *Sporothrix brasiliensis*.

Strain	Glycolipid		Glycan moietiy from glycolipid^a^	
	Mannose^b^	Rhamnose^b^	Mannose^b^	Rhamnose^b^
*Sporothrix schenckii*				
1099-18 ATCC MYA 4821	124.0 ± 11.6	57.1 ± 19.2	116.9 ± 16.5	61.2 ± 10.5
HSS67	117.2 ± 14.6	60.2 ± 20.1	121.5 ± 17.9	57.6 ± 15.6
HSS68	124.6 ± 17.6	59.6 ± 11.5	123.5 ± 21.5	56.6 ± 10.8
HSS69	126.5 ± 9.7	0.1 ± 0.1^*^	118.6 ± 17.8	0.1 ± 0.1^*^
HSS70	128.4 ± 23.1	0.1 ± 0.1^*^	119.8 ± 23.7	0.2 ± 0.2^*^
HSS71	135.3 ± 19.6	0.2 ± 0.1^*^	121.6 ± 17.8	0.1 ± 0.1^*^
HSS72	127.2 ± 17.2	63.4 ± 17.3	121.9 ± 22.3	58.3 ± 22.4
HSS73	120.6 ± 10.5	74.2 ± 21.3	122.6 ± 18.4	62.3 ± 14.6
15.9-7.8,-1.4498.8ptHSS74	134.3 ± 20.5	59.3 ± 12.5	118.3 ± 11.9	57.8 ± 18.8
*Sporothrix brasiliensis*				
5110 ATCC MYA 4823	192.2 ± 26.9	98.8 ± 22.5	186.3 ± 19.8	102.5 ± 27.6
HSB28	196.3 ± 15.6	99.6 ± 19.6	190.8 ± 22.4	102.7 ± 25.6
HSB29	201.3 ± 22.2	102.2 ± 28.6	196.2 ± 27.9	94.5 ± 17.9
HSB35	193.5 ± 29.4	0.2 ± 0.1^*^	188.6 ± 35.6	0.1 ± 0.1^*^
HSB36	198.6 ± 17.8	0.1 ± 0.1^*^	196.0 ± 16.8	0.2 ± 0.2^*^
HSB37	204.6 ± 20.3	0.2 ± 0.2^*^	192.3 ± 18.7	0.2 ± 0.2^*^
HSB38	191.3 ± 11.6	101.4 ± 22.6	195.2 ± 28.6	96.2 ± 28.9
HSB39	196.7 ± 18.6	92.7 ± 18.6	198.6 ± 22.6	98.3 ± 14.6
HSB40	201.4 ± 17.7	99.6 ± 11.8	193.5 ± 27.6	90.6 ± 27.8

^a^Cell wall glycolipid was treated with 1 N KOH to release the glycan moiety.

^b^Reported as μg monosaccharide mg dry cell wall^−1^. Data are means ± SD of three independent experiments performed in duplicate. ^*^P < 0.05 when compared to WT, or control strains (HSS67 and HSS68 for *S. schenckii*; HSB28 and HSB29 for *S. brasiliensis*).

### 3.3 *RHT1* or *RHT2*-silencing affected cell adhesion and biofilm formation in *Sporothrix schenckii* and *Sporothrix brasiliensis*

**Figure 5:**
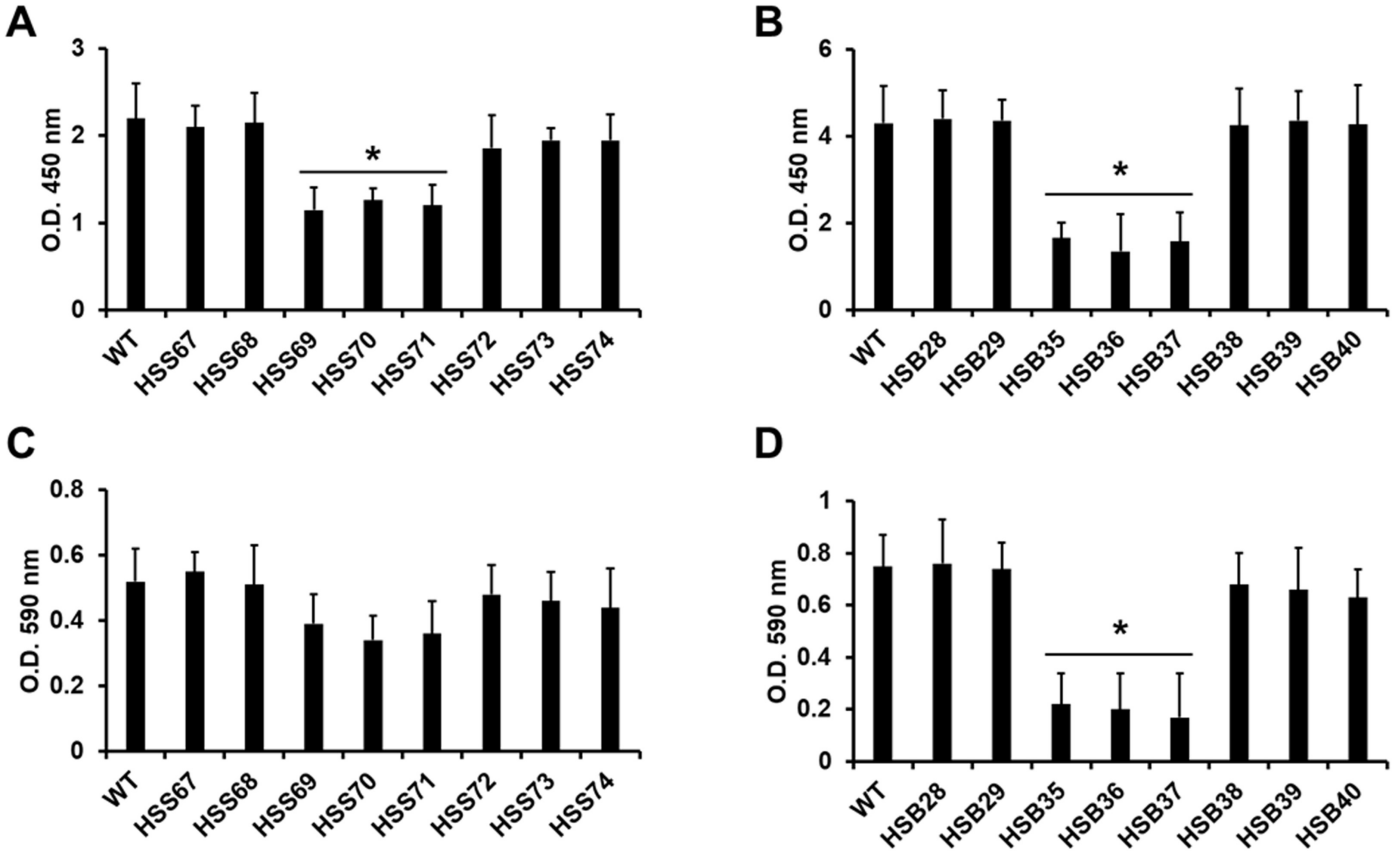
Biofilm formation and adhesion of *Sporothrix schenckii* and *Sporothrix brasiliensis* wild-type, control, *RHT1*-silenced, and *RHT2*-silenced strains to HeLa cells. Plastic microplates were seeded with HeLa cells, and yeast-like cells and cell-cell interactions were incubated for 1 h at 37 °C. Yeast-like cells were labeled with anti-*S. schenckii* Hsp60 antibodies and antibody binding were indirectly detected by a peroxidase-*o*-phenylenediamine system. In **(A)**, results with *S. schenckii* cells, and in **(B)**, results with *S. brasiliensis* cells. In **(C, D)**, microplates were coated with yeast-like cells, non-adherent cells were removed, and biofilms were matured for 24 h at 37 °C. Cell biomass was estimated by crystal violet staining. In **(C)**, results with *S. schenckii* cells, and in **(D)**, results with *S. brasiliensis* cells. Results are shown as means ± SD of three biological replicates and were analyzed with Dunnett's test and then the T-test. *P < 0.05 when compared to WT or control strains (HSS67 and HSS68 for *S. schenckii*; HSB28 and HSB29 for *S. brasiliensis*). For *S. schenckii*, WT is strain 1099-18 ATCC MYA 4821. For *S. brasiliensis*, WT is strain 5110 ATCC MYA 4823.

Next, we assessed whether the silencing of *RHT1* or *RHT2* affected the adhesive properties of *S. schenckii* and *S. brasiliensis* yeast-like cells. An ELISA-based methodology was followed to assess the ability of fungal cells to adhere to extracellular matrix components (García-Carnero et al., 2021). None of the silenced strains used in this study showed changes in the ability to adhere to human fibronectin, thrombospondin-1, type-I collagen, laminin, elastin, fibrinogen, or bovine type-II collagen when compared to the WT or control strains (data not shown). When a similar assay was performed but using the HeLa cell line, a reduction in the ability to adhere to these cells was observed for the *RHT1*-silenced strains from both species (HSS69-HSS71 and HSB35 and HSB37; Figure 5). Contrastingly, the adhesion of the *RHT2*-silenced mutants from both *S. schenckii* and *S. brasiliensis* was similar to that displayed by the WT and control cells (Figure 5). The reduction in adhesion to HeLa cells was more pronounced in the *S. brasiliensis RHT1*-silenced strains than the counterparts in *S. schenckii*, displaying on average a 65% vs. a 45 % reduction in adhesion, respectively.

The ability to form biofilms was also tested in the silenced strains from both species. For *S. schenckii*, despite showing a tendency to reduced ability to form biofilms in the *RHT1*-silenced strains (HSS69-HSS71), this was not statistically significant when compared to WT or control strains (*P* > 0.05 when compared to WT or control strains; Figure 5). For *S. brasiliensis*, the *RHT2* silencing did not affect the ability of yeast-like cells to form biofilms, but *RHT1*-silenced mutants (HSB35-HSB37) showed a significantly reduced ability to form biofilms (Figure 5). These data suggest that *RHT2* has a minor contribution to the adhesion of *S. schenckii* and *S. brasiliensis* to different ligands, and that full-length cell wall glycolipids are required for proper biofilm formation and adhesion to HeLa cells by *S. brasiliensis*. In *S. schenckii, RHT1* has only a significant contribution to binding HeLa cells.

### 3.4 Silencing of *Sporothrix schenckii* and *Sporothrix brasiliensis RHT1* and *RHT2* affected the cytokine production by human peripheral blood mononuclear cells and phagocytosis by human monocyte-derived macrophages

Next, we analyzed whether cell wall rhamnose reduction affected the *S. schenckii* and *S. brasiliensis* interaction with human PBMCs, as depletion of cell wall rhamnose has been reported to affect this interaction (Tamez-Castrellón et al., 2021). We focused on the production of TNFα, IL-6, IL-1β, IL-17, IL-22, and IL-10, as these immunological effectors are good readouts of the consequences of cell wall changes in the *Sporothrix*-host interaction (Tamez-Castrellón et al., 2021; García-Carnero et al., 2023).

**Figure 6:**
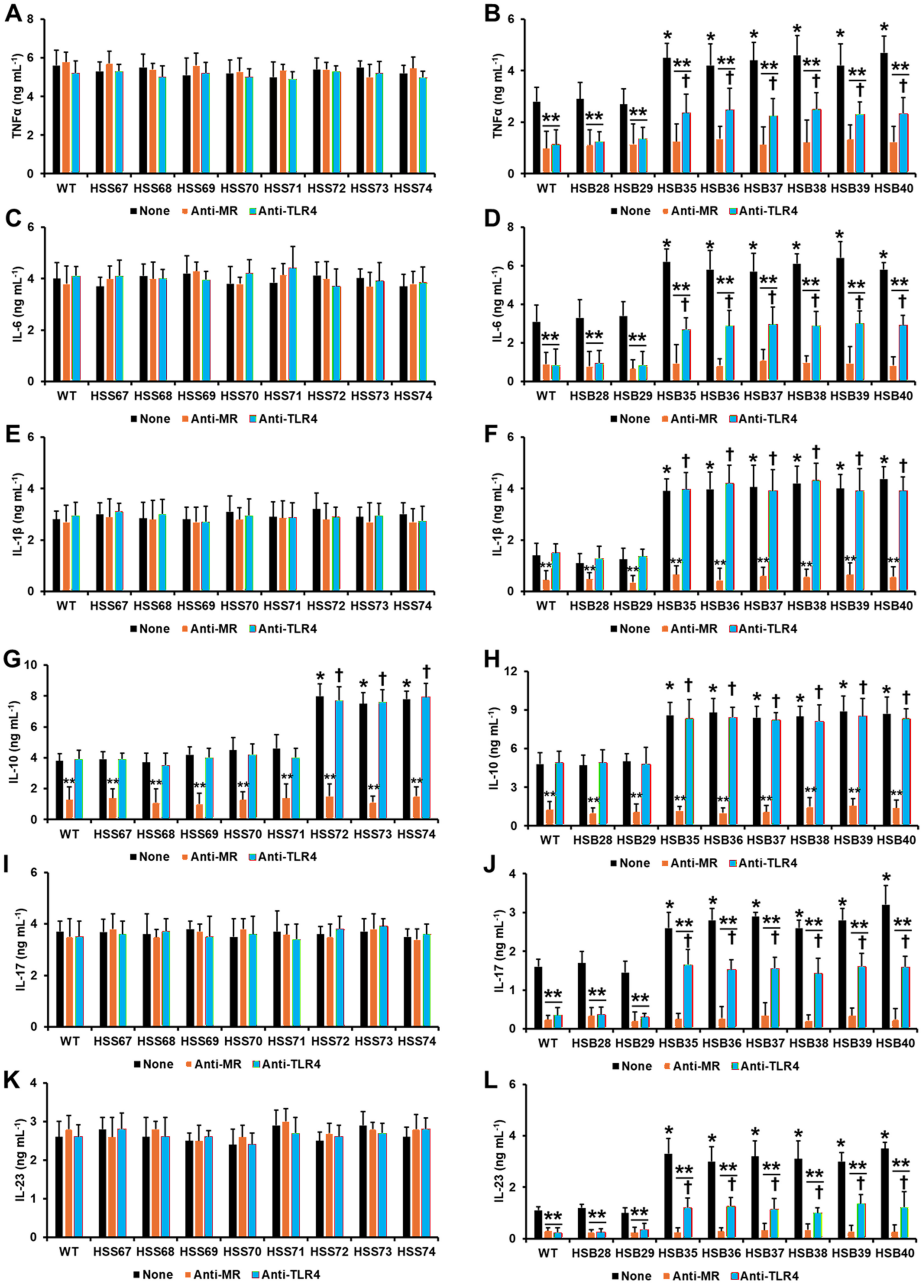
Cytokine stimulation by human peripheral blood mononuclear cell-*Sporothrix* yeast-like cell interaction. Yeast-like cells and human peripheral blood mononuclear cells were coincubated for 24 h at 37 °C **(A–H)** or for 7 days at 37 °C **(I–L)**. Microplates were centrifuged, the supernatants saved, and used to quantify cytokines by ELISA. None, PBMCs were preincubated only with culture medium; Anti-MR, PBMCs were preincubated with anti-mannose receptor antibody; Anti-TLR4, PBMCS were preincubated with anti-TLR4 4 antibody. Data are means ± SD obtained with samples from eight healthy donors. Results were analyzed with Dunnett's test and then the Mann-Whitney U-test. *P < 0.05 when compared to wild-type (WT) or control cells (HSS67 and HSS68, or HSB28 and HSB35). **P < 0.05 when compared to None cells from the same strain; ^†^*P* < 0.05 when compared to WT or control cells under the same treatment. For *S. schenckii*, WT is strain 1099-18 ATCC MYA 4821. For *S. brasiliensis*, WT is strain 5110 ATCC MYA 4823.

Both *S. schenckii* and *S. brasiliensi*s WT strains showed similar cytokine profiles to those of the control strains, indicating that the binary vector backbone did not affect the cell-cell interaction (Figure 6). In the case of *S. schenckii, RHT1*- or *RHT2*-silenced mutants did not affect TNF, IL-6, IL-1β, IL-17, or IL-23 production (Figure 6). In the case of IL-10 stimulation, only the *RHT2* silencing positively affected cytokine stimulation (Figure 6). Contrastingly, silencing of *RHT1* or *RHT2* in *S. brasiliensis* positively affected the production of TNFα, IL-6, IL-1β, IL-10, IL-17, and IL-23 at the same level in both sets of silencing mutants (Figure 6).

We also analyzed the contribution of some pattern recognition receptors (PRRs) in this interaction. We used laminarin to block dectin-1-β-1,3-glucan interaction (Martínez-Álvarez et al., 2017), but no significant changes were observed among the WT and silenced strains (data not shown). A similar trend was observed when TLR2 or CR3 were antibody-directed blocked (data not shown). The antibody-directed blocking of MR or TLR4 did not affect TNFα, IL-6, IL-1β, IL-17, or IL-23 stimulation by *S. schenckii* WT, control, or mutant strains (Figure 6). However, for IL-10 production, MR blocking significantly reduced IL-10 production stimulated by the WT, control, or silenced strains (Figure 6). No significant changes were observed with cells preincubated with anti-TLR4 antibody, though (Figure 6).

For *S. brasiliensis*, WT and control cells stimulated significantly reduced levels of TNFα, IL-6, IL-17, and IL-23 when human PBMCs were preincubated with either anti-MR or anti-TLR4 antibody (Figure 6). For IL-10 and IL-1β stimulation, only the MR blocking significantly reduced the cytokine levels (Figure 6). For *RHT1* and *RHT2*-silenced mutants, MR blocking significantly reduced the production of all the analyzed cytokines (Figure 6). For TNFα, IL-6, IL-17, and IL-23 production, a similar trend was observed with cell-cell interactions in the presence of anti-TLR4 antibody, but cytokine levels were higher when compared to those stimulated by WT cells interacting with TLR4-blocked cells (Figure 6). In contrast, IL-1β and IL-10 stimulation by *RHT1*- and *RHT2*-silenced mutants was insensitive to the presence of the anti-TLR4 antibody (Figure 6). In all cases, control interactions with irrelevant isotype-matched antibodies gave similar results to interactions with specific antibodies (data not shown).

**Figure 7:**
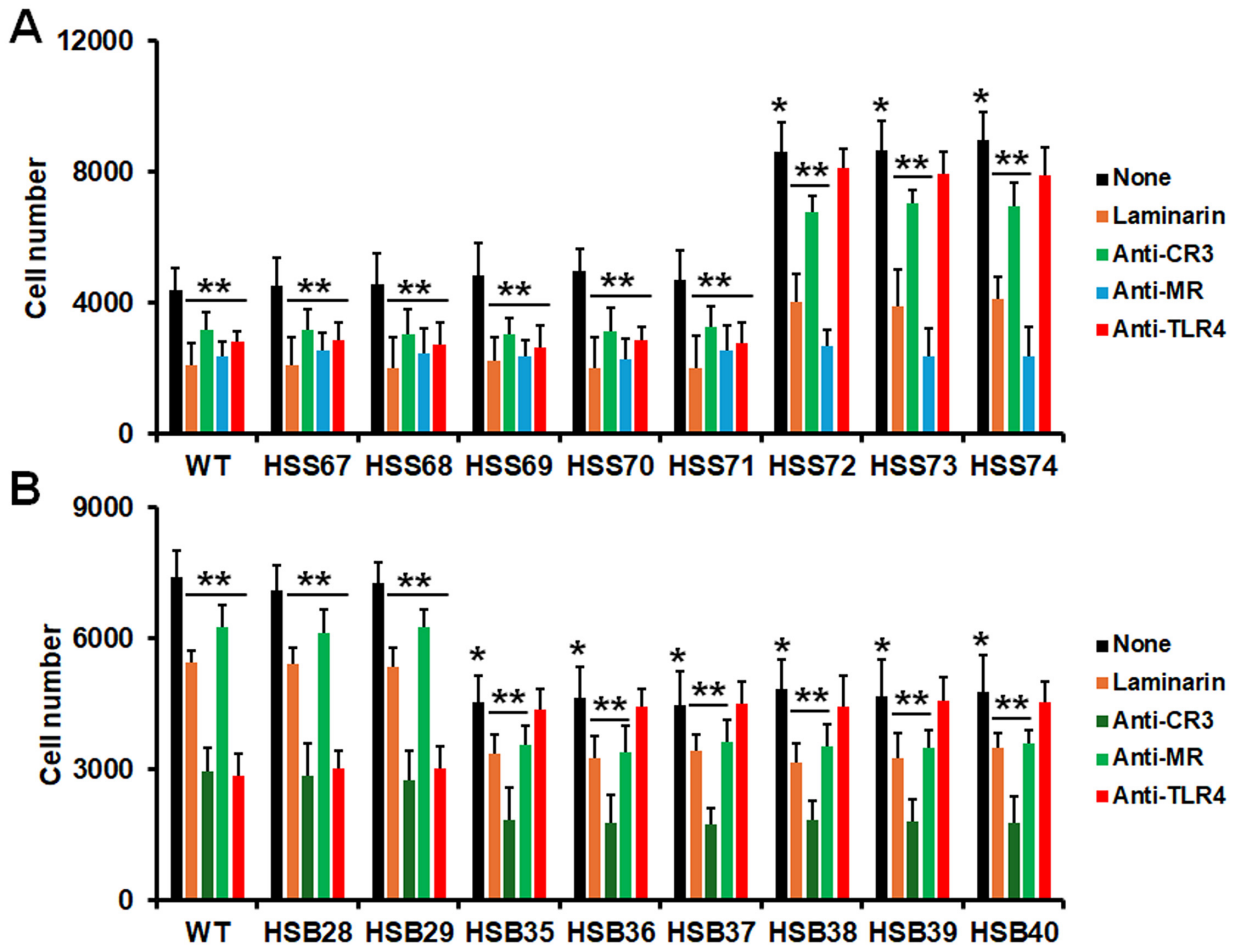
Phagocytosis of *Sporothrix schenckii* and *Sporothrix brasiliensis* yeast-like cells by human monocyte-derived macrophages. Fungus-immune cell interaction was performed for 2 h at 37 °C and 5% (v/v) CO_2_, and the phagocytic process was analyzed by flow cytometry with acridine-orange-labeled yeast-like cells. Only positive cells in the red channel were included in the charts and were considered to be in the late stage of phagocytosis. In **(A)**, interaction of *S. schenckii* strains with human monocyte-derived macrophages. In **(B)**, interactions of *S. brasiliensis* strains with the human immune cells. None, monocyte-derived macrophages were preincubated only with culture medium; Laminarin, immune cells were preincubated with the β-1,3-glucan antagonist laminarin; Anti-CR3, immune cells were preincubated with anti-complement receptor 3 antibody; Anti-MR, immune cells were preincubated with anti-mannose receptor antibody; Anti-TLR4, human cells were preincubated with anti-TLR4 antibody. Data are means ± SD obtained with samples from eight healthy donors. Results were analyzed with Dunnett's test and then the Mann-Whitney U-test. *P < 0.05 when compared to wild-type (WT) or control cells (HSS67 and HSS68, or HSB28 and HSB35). **P < 0.05 when compared to None cells from the same strain. For *S. schenckii*, WT is strain 1099-18 ATCC MYA 4821. For *S. brasiliensis*, WT is strain 5110 ATCC MYA 4823.

Next, we analyzed the ability of human monocyte-derived macrophages to phagocytize the strains under analysis. We only analyzed the uptake of fungal cells in the late stage of phagocytosis, as more than 85% of human cells are in this stage under the utilized experimental conditions (Gómez-Gaviria et al., 2023a,b). For *S. schenckii*, WT and control strains were phagocytosed at the same rate, and β-1,3-glucan, CR3, MR, and TLR4 were relevant PRRs involved in uptake, as antagonist compounds significantly reduced phagocytosis, as reported (Gómez-Gaviria et al., 2023a,b) (Figure 7). The *RHT1*-silenced mutants (HSS69-HSS71) showed a similar phagocytosis rate and similar pattern recognition receptor dependence to the WT and control strains (Figure 7). Contrastingly, the *RHT2*-silenced mutants (HSS72-HSS74) showed increased uptake when interacting with human monocyte-derived macrophages, in a process that was dependent on β-1,3-glucan, CR3, and MR, but not on TLR4 (Figure 7). Immune cells interacting with *S. brasiliensis* WT or control cells showed similar uptake levels, and a strong dependence on CR3 and TLR4, and a moderate contribution of β-1,3-glucans and MR (Figure 7). The *RHT1*- and the *RHT2*-silenced strains (HSB35-HSB37 and HSB38-HSB40, respectively) were less phagocytosed than the WT and control cells with the loss of TLR4 contribution to the process (Figure 7). In all cases, control interactions with irrelevant isotype-matched antibodies gave similar results to interactions with specific antibodies (data not shown).

### 3.5 *RHT1* or *RHT2* silencing and virulence in *Galleria mellonella*

**Figure 8:**
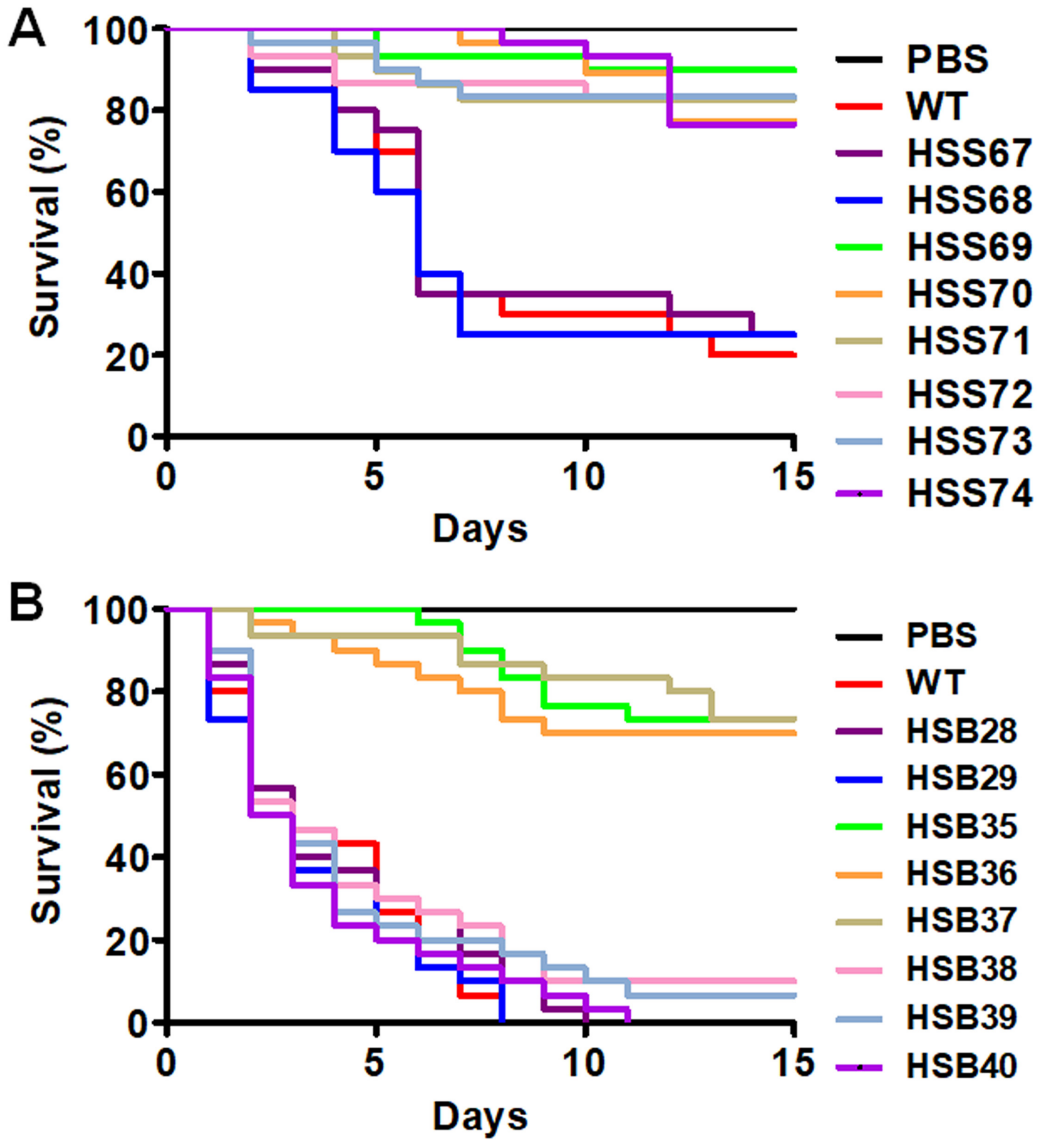
Changes in virulence upon *RHT1* or *RHT2* silencing in *Sporothrix schenckii* or *Sporothrix brasiliensis*. *Galleria mellonella* larvae were divided into groups of 30 individuals, and these were inoculated with the different strains analyzed. The mortality was monitored daily for 2 weeks. PBS, refers to groups inoculated with the buffer PBS to control the inoculation and housing processes. Results are expressed in Kaplan–Meier plots. In **(A)**, results were generated with *S. schenckii* strains (WT, strain 1099-18 ATCC MYA 4821). In **(B)**, results were generated with *S. brasiliensis* strains (WT, strain 5110 ATCC MYA 4823).

The *G. mellonella* larvae, as an experimental model to analyze fungal virulence, have gained momentum in the last decades, because it is an easy-to-breed, keep, and handle model in the laboratory, with less strict ethical considerations than vertebrates. Moreover, it can reproduce the virulence results obtained in mice and other rodents (Jacobsen, 2014; Clavijo-Giraldo et al., 2016; Vargas-Macías et al., 2022). The larva group inoculated with *S. schenckii* WT strain and the control group HSS67 and HSS68 strains showed similar survival curves with about 70% of larva death and a median survival of 6.0 ± 0.5 days (Figure 8). Contrastingly, groups inoculated with either the *RHT1* or the *RHT2*-silenced mutants showed similar killing curves, with 17.0 ± 6.0% of dead animals and a median survival of more than 15 days (Figure 8). For *S. brasiliensis*, the WT and control (HSB28 and HSB29) strains showed similar killing curves, with no animals alive after the inoculation and a median survival of 3.0 ± 0.5 days (Figure 8). The *RHT2*-silenced strains (HSB38-HSB40) showed similar killing curves, with 8.0 ± 2.0 % of alive animals at the end of the observation period and a median survival of 3.0 ± 0.5 days (Figure 8). The *RHT1*-silenced mutants (HSB35-HSB37) showed lower ability to kill *G. mellonella*, with 28.0 ± 2.0 % of dead animals at the end of the observation period and a median survival of more than 15 days (Figure 8). When the fungal burden was assessed, the control group gave no fungal colony-forming unit (CFU) withdrawn from the hemolymph, while all the analyzed *S. schenckii* strains showed similar CFU in hemolymph (3.1 ± 0.8 × 10 ^5^ CFU). Similarly, the *S. brasiliensis* strains showed an average of 3.5 ± 0.6 × 10^5^ CFU, suggesting that all the analyzed strains showed a similar ability to grow within the insect's hemolymph.

We also measured the cell damage in the hemolymph, defined as cell-free lactate dehydrogenase and referred to here as cytotoxicity (García-Carnero et al., 2020; Lozoya-Pérez et al., 2020). Strains showing low ability to kill *G. mellonella* larvae, named *S. schenckii RHT1*-silenced (HSS69-HSS71) and *RHT2*-silenced (HSS72-HSS74) mutants and *S. brasiliensis RHT1*-silenced mutants (HSB35-HSB37), induced low levels of cytotoxicity in inoculated larvae when compared with the corresponding WT or control strains (Table 3). The hemocyte levels and phenoloxidase activity, two immunological components found in the insect hemolymph (García-Carnero et al., 2020; Lozoya-Pérez et al., 2020), were also affected in these groups of mutants, with diminished hemocyte and phenoloxidase levels when compared to the WT and control strains (Table 3). The *S. brasiliensis RHT2*-silenced strains stimulated changes in cytotoxicity, hemocytes, and phenoloxidase similar to those induced by the WT and control cells (Table 3).

**Table 3:** Cytotoxicity, hemocyte levels, and phenoloxidase activity in *Galleria mellonella* larvae infected with wild-type, control, *RHT1-* or *RHT2*-silenced strains from *Sporothrix schenckii* and *Sporothrix brasiliensis*.

Strain	Cytotoxicity (%)^a^	Hemocytes ( × 10^6^) mL^−1^	Phenoloxidase^b^
PBS^c^	8.4 ± 3.8	3.7 ± 0.4	0.5 ± 0.3
*Sporothrix schenckii*			
1099-18 ATCC MYA 4821	84.6 ± 10.5	8.5 ± 0.7	3.2 ± 0.5
HSS67	94.0 ± 12.4	8.8 ± 0.9	3.4 ± 0.4
HSS68	89.7 ± 11.5	8.6 ±0.8	3.0 ± 0.2
HSS69	32.1 ± 14.1^*^	4.8 ± 0.9^*^	1.0 ± 0.6^*^
HSS70	25.4± 15.6^*^	4.6 ± 1.1^*^	0.8 ± 0.5^*^
HSS71	34.5 ± 12.7^*^	4.2 ± 0.7^*^	0.7 ± 0.3^*^
HSS72	25.6 ± 15.3^*^	4.4 ± 1.0^*^	1.3 ± 0.5^*^
HSS73	22.4 ± 14.7^*^	4.4 ± 0.8^*^	1.2 ± 0.4^*^
15.9-7.8,-1.4498.8ptHSS74	34.2 ± 12.8^*^	4.6 ± 0.9^*^	1.0 ± 0.4^*^
*Sporothrix brasiliensis*			
5110 ATCC MYA 4823	98.5 ± 1.4	9.7 ± 1.2	4.1 ± 0.8
HSB28	99.4 ± 1.1	9.5 ±1.2	4.5 ± 0.9
HSB29	97.3 ± 2.5	9.8 ± 0.6	4.4 ± 0.9
HSB35	22.7 ± 18.6^*^	5.2 ± 1.0^*^	1.5 ± 0.6^*^
HSB36	27.5 ± 9.4^*^	4.8 ± 0.8^*^	1.2 ± 0.9^*^
HSB37	22.4 ± 11.8^*^	4.9 ± 0.6^*^	1.1 ± 0.5^*^
HSB38	94.5 ± 16.5^*^	9.7 ± 1.2	4.3 ± 0.9
HSB39	94.6 ± 8.9^*^	9.6 ± 1.1	4.2 ± 0.4
HSB40	97.8 ± 17.4^*^	9.7 ± 0.6	4.3 ± 0.5

^a^Cell-free hemolymph was collected and used to measure lactate dehydrogenase activity. Data are normalized to the enzyme activity shown by lysed hemocytes (referred to as 100%).

^b^Expressed as the Δ_490nm_ min^−1^ μg protein ^−1^.

^c^Larvae inoculated only with PBS.

In *S. schenckii*, ^*^P < 0.05 when compared with the values obtained in animals infected with strain 1099-18 ATCC MYA 4821, HSS67 or HSS68. In *S. brasiliensis*, ^*^P < 0.05 when compared with the values obtained in animals infected with strain 5110 ATCC MYA 4823, HSB28, or HSB29.

## 4 Discussion

Rhamnose is one of the most distinctive components of the cell wall of pathogenic *Sporothrix* species and has been associated with key processes involved in host–pathogen interactions, including immune recognition, phagocytosis, and virulence (Tamez-Castrellón et al., 2021). Although previous studies have demonstrated the biological relevance of this monosaccharide and of peptidorhamnomannan as one of the major surface glycoconjugates, the mechanisms responsible for the incorporation of rhamnose into the different cell wall components remained poorly characterized (Figueiredo et al., 2010; Tamez-Castrellón et al., 2021). In the present study, we investigated for the first time the function of two rhamnosyltransferases, *RHT1* and *RHT2*, in *S. schenckii* and *S. brasiliensis*, demonstrating that both enzymes play specialized and non-redundant roles in the biosynthesis of rhamnose-containing glycoconjugates.

Although silencing of *RHT1* and *RHT2* resulted in a comparable reduction in overall rhamnosyltransferase activity, the effects on cell wall composition were markedly different. Whereas, *RHT2* silencing selectively decreased the rhamnose content of *N-*linked and *O-*linked glycans, *RHT1* silencing primarily altered the carbohydrate composition of a surface glycolipid. Collectively, these findings indicate that both enzymes perform specialized functions and suggest that the incorporation of rhamnose into glycoproteins and glycolipids occurs through independent biosynthetic pathways. This functional specialization provides a new perspective on the organization of *Sporothrix* glycoconjugates. Previous studies focused on the UDP-rhamnose biosynthetic pathway demonstrated that a global reduction in this sugar affects cell wall composition, immune recognition, and virulence; however, these studies were unable to determine which rhamnosylated structures were responsible for the observed phenotypes (Figueiredo et al., 2010; Tamez-Castrellón et al., 2021). The findings presented here help address this question and suggest that distinct rhamnose-containing glycoconjugates make differential contributions to host–pathogen interactions.

The functional specialization observed for *RHT1* and *RHT2* is particularly noteworthy because cell wall rhamnose is uncommon among fungi and has been reported in only a limited number of species, including *Sporothrix* spp., *P. boydii*, and several phytopathogenic fungi (Chávez-Santiago et al., 2025). In these organisms, rhamnose-containing glycoconjugates have repeatedly been associated with host interaction and pathogenicity. Therefore, the identification of distinct rhamnosylation pathways in *Sporothrix* may represent a broader biological principle governing the organization and function of rhamnose-containing glycoconjugates in fungal pathogens.

The involvement of *RHT2* in the incorporation of rhamnose into *N-*linked and *O-*linked glycans is consistent with the notion that cell wall glycoproteins represent one of the major reservoirs of this monosaccharide in pathogenic *Sporothrix* species. Given that peptidorhamnomannan is one of the most abundant and best-characterized surface glycoconjugates (Bezerra, 2011), *RHT2* likely contributes to the biosynthesis of rhamnose-containing surface glycoproteins, potentially including components associated with peptidorhamnomannan.

In contrast, the identification of alterations in a surface glycolipid dependent on *RHT1* reveals an additional level of complexity in *Sporothrix* glycobiology. Although the presence of cell wall glycolipids has been previously reported in this genus, the biological functions of these molecules remain poorly understood (Carlos et al., 2003, 2009; Martínez-Álvarez et al., 2014). Our results suggest that glycolipids also represent important reservoirs of surface-exposed rhamnose and that their biosynthesis depends on mechanisms distinct from those involved in glycoprotein modification. This observation significantly expands the current understanding of rhamnose distribution on the fungal cell surface and raises the possibility that different rhamnose-containing glycoconjugates perform specialized functions during host–pathogen interactions. Notably, previous studies demonstrated that lipid fractions isolated from *S. schenckii* inhibit macrophage phagocytosis and stimulate inflammatory responses (Carlos et al., 2003), indicating that *Sporothrix* glycolipids possess biological activity during host interaction. The present findings extend these observations by suggesting that rhamnose-containing glycolipids are among the glycolipid species contributing to these virulence-associated functions.

The biological relevance of this functional specialization became evident when processes associated with the early stages of host interaction were examined. In both species, *RHT1* silencing significantly impaired adhesion to HeLa cells and reduced biofilm formation, whereas *RHT2* silencing produced less pronounced or no detectable effects on these phenotypes. These findings suggest that *RHT1*-dependent rhamnosylated glycolipids play an important role in cell surface organization and in the establishment of cell–cell and cell–substrate interactions. The involvement of glycolipids in adhesion-related processes has been described in several pathogenic microorganisms (Toledo et al., 2001; Suzuki et al., 2008; Diederich et al., 2014; Watchaputi et al., 2025), where these molecules contribute to the physicochemical properties of the cell surface, hydrophobicity, and interactions with host components. Although the mechanisms underlying these processes in *Sporothrix* remain unknown, the selective loss of rhamnose observed in the *RHT1*-silenced mutants suggests that this monosaccharide may be part of structures required to maintain proper surface organization and promote the initial stages of host colonization. Notably, these effects were more pronounced in *S. brasiliensis* than in *S. schenckii*. This observation is consistent with the greater adhesive capacity, biofilm-forming ability, and virulence that characterize *S. brasiliensis* compared with other members of the pathogenic clade (Arrillaga-Moncrieff et al., 2009; Padró-Villegas et al., 2025, 2026), suggesting that rhamnosylated glycolipids may make a particularly important contribution to the mechanisms that facilitate infection establishment and progression in this species.

Although the biosynthetic functions of *RHT1* and *RHT2* appear to be conserved in *S. schenckii* and *S. brasiliensis*, the biological consequences resulting from the alteration of these glycoconjugates were markedly different between the two species. This observation suggests that, in addition to differences in cell wall composition, the organization and functional contribution of rhamnose-containing structures may vary among closely related species within the pathogenic *Sporothrix* clade, as already reported at the genomic, proteomic, and glycobiological level (Teixeira et al., 2014; Lopes-Bezerra et al., 2018a,b; Silva-Bailão et al., 2021).

Species-specific differences were particularly evident during interactions with human immune cells. In *S. schenckii*, the alterations associated with *RHT2* silencing had a more pronounced impact on cytokine production than those observed in *RHT1*-silenced mutants. Given that *RHT2* is primarily involved in the incorporation of rhamnose into *N-*linked and *O-*linked glycans, these findings suggest that rhamnosylated glycoproteins constitute the main determinants involved in immune recognition in this species, consistent with previous studies identifying peptidorhamnomannan as one of the major immunologically relevant ligands of *S. schenckii* (Bezerra, 2011; García-Carnero et al., 2021; Neves et al., 2021). In contrast, both *RHT1*- and *RHT2*-silenced mutants of *S. brasiliensis* displayed significant alterations in cytokine production, indicating that both rhamnosylated glycoproteins and glycolipids contribute to immune activation in this species.

A particularly remarkable finding was the altered contribution of TLR4 to the recognition of the silenced mutants. Previous studies demonstrated that a global reduction in surface rhamnose, achieved through disruption of UDP-rhamnose biosynthesis, significantly decreases the dependence on TLR4 during the interaction between *S. schenckii* and human immune cells (Tamez-Castrellón et al., 2021). However, this study was unable to identify the specific glycoconjugates responsible for this phenomenon. The results presented here extend this model by demonstrating that the contribution of TLR4 depends on both the affected glycoconjugate and the species under investigation. In *S. brasiliensis*, silencing of either *RHT1* or *RHT2* reduced TLR4-dependent cytokine production and impaired the contribution of this receptor to phagocytosis, suggesting that both glycoproteins and glycolipids are ligands recognized by TLR4. In contrast, in *S. schenckii*, the effects associated with *RHT2* silencing had a more pronounced impact on the immune-related phenotypes analyzed, further supporting the notion that rhamnosylated glycoproteins represent the major immune recognition determinants in this species. Collectively, these findings indicate that TLR4 activation is determined not only by the presence of rhamnose itself, but also by the molecular context in which this monosaccharide is displayed on the fungal cell surface.

The differences between the two species were also evident during the phagocytic process. While *S. schenckii RHT2*-silenced mutants showed increased uptake by macrophages, both *RHT1*- and *RHT2*-silenced mutants of *S. brasiliensis* displayed a significant reduction in phagocytosis. One possible explanation for the increased uptake observed in *S. schenckii* is that reduced glycoprotein rhamnosylation leads to a reorganization of the cell surface that favors the exposure of other immunologically relevant ligands, such as mannose-rich residues. In contrast, the results obtained with *S. brasiliensis* suggest that rhamnose-containing glycoconjugates participate more directly in phagocyte recognition, such that their alteration decreases the efficiency of cellular uptake. Collectively, these findings highlight that the contribution of rhamnose to phagocyte interactions is more complex than previously proposed and depends not only on the nature of the affected glycoconjugate but also on the biological context of each species.

The biological importance of *RHT1*-dependent rhamnosylated glycolipids became particularly evident in the virulence assays. In both species, *RHT1*-silenced mutants exhibited defects in adhesion and biofilm formation; however, these alterations had distinct consequences for pathogenicity. Interestingly, in *S. schenckii*, silencing of either *RHT1* or *RHT2* significantly reduced virulence in *G. mellonella*, suggesting that both rhamnosylated glycoproteins and glycolipids contribute to the pathogenic potential of this species. This behavior contrasts with that observed in *S. brasiliensis*, where the predominant effect was associated with alterations in *RHT1*-dependent glycolipids. In this species, *RHT1* silencing resulted in a marked reduction in virulence, whereas *RHT2* silencing had only a limited effect on this phenotype, suggesting that rhamnosylated glycolipids play a particularly important role in the pathogenicity of *S. brasiliensis*. Collectively, these findings indicate that the rhamnosylated glycolipids synthesized by *RHT1* represent important virulence determinants, particularly in *S. brasiliensis*, although they do not exclude an additional contribution from other rhamnose-containing glycoconjugates.

Finally, our results demonstrate that *RHT1* and *RHT2* perform specialized functions in the biosynthesis of rhamnose-containing glycoconjugates in *Sporothrix* and reveal that the biological contribution of these structures differs between *S. schenckii* and *S. brasiliensis*. The data indicate that rhamnose does not act as a uniform determinant of host–pathogen interactions; rather, it exerts distinct biological functions depending on the glycoconjugate to which it is linked. While rhamnosylated glycoproteins appear to participate predominantly in immune recognition and the activation of TLR4-dependent responses, *RHT1*-dependent glycolipids contribute more substantially to cell surface organization, adhesion, biofilm formation, and virulence. Although enzymatic digestion and monosaccharide analyses strongly support the proposed composition of the glycolipid glycan moiety, definitive structural characterization will require mass spectrometry and NMR approaches. These findings significantly expand the current understanding of *Sporothrix* glycobiology and provide new evidence that different rhamnose-containing glycoconjugates contribute in a coordinated, yet non-equivalent, manner to the biology and pathogenicity of species within the pathogenic clade. Consequently, this work proposes a new conceptual framework for understanding how the distribution of rhamnose among distinct glycoconjugates, not only its abundance, determines specific functions during the interaction between *Sporothrix* and the host, and how these contributions may differ even between closely related species of the pathogenic clade.

## Data Availability

The raw data supporting the conclusions of this article will be made available by the authors, without undue reservation.
